# Antiproliferative Ruthenium Complexes Containing Curcuminoid Ligands Tested In Vitro on Human Ovarian Tumor Cell Line A2780, towards Their Capability to Modulate the NF-*κ*BTranscription Factor, FGF-2 Growth Factor, and MMP-9 Pathway

**DOI:** 10.3390/molecules27144565

**Published:** 2022-07-18

**Authors:** Janka Leskovská, Natalia Miklášová, Paul Milan Kubelac, Patriciu Achimaş-Cadariu, Jindra Valentová, Mário Markuliak, Eva Fischer-Fodor

**Affiliations:** 1 Department of Chemical Theory of Drugs, Faculty of Pharmacy, Comenius University in Bratislava, Kalinčiakova 8, 83104 Bratislava, Slovakia; valentova@fpharm.uniba.sk (J.V.); markuliak@fpharm.uniba.sk (M.M.); 2 Institute of Oncology “Prof.Dr.I.Chiricuta”, RO-400015 Cluj-Napoca, Romania; paulkubelac@yahoo.com (P.M.K.); patrick.achimas@hotmail.com (P.A.-C.); fischer.eva@iocn.ro (E.F.-F.); 3 Department of Oncology, “Iuliu Hatieganu” University of Medicine and Pharmacy, RO-400012 Cluj-Napoca, Romania; 4 Department of Oncological Surgery and Gynecological Oncology, “Iuliu Hatieganu” University of Medicine and Pharmacy, RO-400012 Cluj-Napoca, Romania

**Keywords:** bisdemethoxycurcumin (BDMC), syringaldehyde curcumin, ruthenium(II) complexes, cytotoxicity, NF-*κ*B, FGF-2, MMP-9

## Abstract

So far, the polyphenolic components of turmeric have shown a significant pharmacological preventative activity for a wide spectrum of diseases, including oncological disorders. This type of natural product could be of great interest for the inhibition of cancer cell proliferation, displaying less side effects in comparison to classical chemotherapeutics. The poor bioavailability and quick metabolism of such natural compounds require new investigative methods to improve their stability in the organisms. A synthetic approach to increase the efficiency of curcuminoids is to coordinate them to metals through the beta-dicarbonyl moiety. We report the synthesis and the biological attempts on human ovarian carcinoma A2780 of ruthenium(II) complexes **1**–**4**, containing curcuminoid ligands. The cytotoxicity of complexes **1**–**4** proves their antiproliferative capability, and a correlation between the IC_50_ values and NF-*κ*B transcription factor, FGF-2, and MMP-9 levels was figured out through the principal component analysis (PCA).

## 1. Introduction

Curcumin, used for hundreds of years in Indian and Ayurvedic medicine, is obtained from the plant *Curcuma longa* L., Zingiberaceae [1,2]. This natural polyphenolic molecule exhibits numerous biological activities, including antithrombotic [3], anti-inflammatory [4], immunomodulatory [5], antioxidant [6], antiarthritic [7], plumoprotective, lipid-modifying [8], antidiabetic [9], hepatoprotective [10], nephroprotective [11] and anticancer [12] properties. The antitumor effect of curcumin acts in various stages of carcinogenesis, including the prevention of cancer inflammation, inhibition of the activation of oncogenes, induction of apoptosis, inhibition of proliferation of cancer cells, prevention of metastasis, and sensibilization of cancer cells on chemotherapy [13]. Despite the mentioned beneficial effects, there are some disadvantages to these phytochemicals (weak absorption, low bioavailability, rapid metabolism, and fast systemic elimination [14]), which prevent their clinical use. These drawbacks can be overcome by designing new drugs with modulated pharmacological and biological properties with superior antitumor activity and lower toxicity compared to recent treatments.

Towards the three main constituents of turmeric, curcumin (CUR), demethoxycurcumin (DMC), and bisdemethoxycurcumin (BDMC), the latest curcuminoid possesses multiple mechanisms of action. BDMC inhibits cell proliferation, invasion and migration, metastasis, and tumor growth and induces apoptosis in tumor cells, and generates ROS levels in breast cancer, lung cancer, gastric cancer, and ovarian cancer [15,16]. It may act also as a potential novel antimetastatic agent for the treatment of human cervical cancer by suppressing migration and invasion of HeLa cells via inhibition of NF-*κ*B, MMP-2, and -9 pathways [17,18]. BDMC can significantly alleviate in vivo nephrotoxicity caused by cisplatin through anti-oxidant and anti-inflammatory effects and has a synergistic anti-cancer effect with cisplatin [19].

To increase the pharmacological effects of natural compounds such as curcumin, alongside the use of nanoparticles, liposomes, phospholipid complexes, and micelles, the complexation of curcumin with transition metal ions enhances the bioavailability and solubility in water and improves the pharmacodynamic effects of curcumin. The capability of curcumin-like ligands to coordinate to metal centers and to form complexes with improved properties might be one of the pathways to lower the beforementioned disadvantages of these compounds. Metal complexes of curcuminoids exhibit higher stability under physiological conditions and easy detection in vivo, showing a broad potential in molecular imaging and anticancer strategies [20,21]. The highly conjugated *β*-diketone moiety found in the chemical structure of curcumin can readily form metal chelates of type 1:1 and 1:2 with several metal ions with divalent and trivalent inorganic molecules like Mn^2+^, Fe^2+^, Cu^2+^, Zn^2+^ and Ru^2+^ [22]. Over the past decade, the complexation of curcumin with metal ions was reported to be one of the most practical approaches for the evaluation of pharmacological effects of curcumin, like anticancer, antioxidant, antimicrobial, and anti-inflammatory properties [23]. Additionally, the chelates of curcuminoids with various kinds of metals could increase photostability, phototherapeutic capability, and pro-apoptotic effect [24]. Metal-based complexes of curcuminoids not only improved the biological activity but also reduced the original toxicity of curcuminoids [4]. Previously synthesized ruthenium complexes with BDMC showed increased cytotoxicity in breast cancer cell line [25] and bifunctional ruthenium(II) polypyridyl complexes of curcumin has shown potential anticancer activities by interacting with DNA and MEK/ERK signaling pathway as well as DNA intercalation [26] and therefore further research of such molecules should be the subject of the new study.

In the development of new metal-centered complexes designed for precision therapy, an important role is played by ruthenium compounds [27], which can act as good alternatives to standard platinum-based drugs [28]. Several reports about the effectiveness of ruthenium complexes against gastrointestinal tumors [29], and breast cancer cell lines in vitro, have been published. Certain ruthenium complexes are more selective than cisplatin [30], having the capacity to target several genes in ovarian cancer cells [31], including those responsible for the resistance of the A2780 tumor to platinum-based standard drugs. These complexes may be effective even against platinum drug-resistant cancer cells [28]. The central metal modulates the mechanism of action of the metallodrug [32], but the multifaceted metal-ligand interaction is the basis of drug function, therefore improving the ligand could be an efficient structure-based design for novel Ru(II) complexes. It is noteworthy that curcumin derivates themselves are good inhibitors against tumor cell growth [33,34] and might serve as ligands in antitumor metal complexes [35]. Bipyridyl-like derivatives are used in syntheses to improve the cytotoxicity of metal-based complexes forming weak and reversible interactions towards DNA during the DNA self-repair processes [36].

Several studies of Ru(II) complexes with bipyridyl [37] revealed the antitumor properties of such compounds. One of the studies on biological systems has been done on the A2780 cell population [38]. However, the cytotoxicity of the compound was modest. The Ru(II) complex with dimethyl-bipyridyl exhibited certain toxicity [39], and therefore it was utilized as a chelating ligand or embedded in nanostructures [40]. Further investigations used both mentioned complexes mainly as a precursor for more potent compounds.

The present paper reports the synthesis, characterization, and determination of cytotoxicity for four ruthenium complexes (**1**–**4**) containing bisdemethoxycurcumin (BDMC) ligand **L1** and syringaldehyde curcumin ligand **L2**. Biological tests conducted for complexes **1**–**4** against the A2780 human ovarian adenocarcinoma cell lines, involved the MTT (3-(4,5-dimethylthiazol-2-yl)-2,5-diphenyltetrazolium bromide) assay, the FGF-2 (Soluble basic Fibroblast Growth Factor production), the MMP-9 (Matrix metalloproteinase-9), and the intracellular nuclear factor kappa-light-chain-enhancer of activated B cells (NF-*κ*B).

## 2. Results and Discussion

### 2.1. Synthesis and Structural Characterization of Ruthenium Complexes

**Figure 1:**
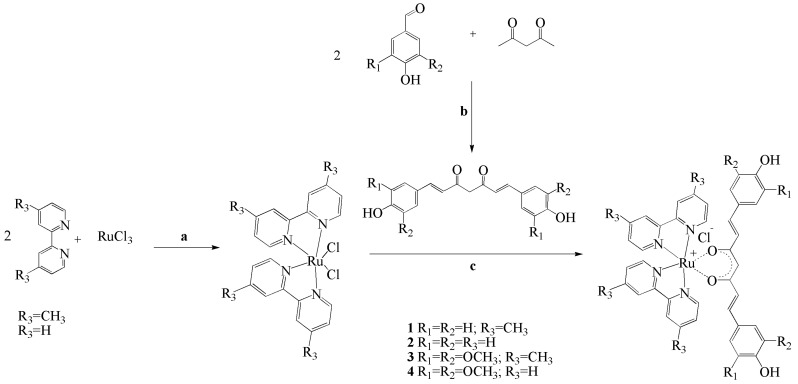
Scheme of preparation of ruthenium complexes with bisdemethoxycurcumin and syringaldehyde curcumin; **a**—DMF, 5 h reflux, **b**—B_2_O_3_, (nBuO)_3_B, nBuNH_2_, HCl, **c**—DMF/H_2_O, Na_2_CO_3_, 3 h reflux.

Four ruthenium complexes **1**–**4**, have been synthesized (Figure 1), structurally characterized, and biologically tested on the human carcinoma A2780 cell line. 

**Figure 2:**
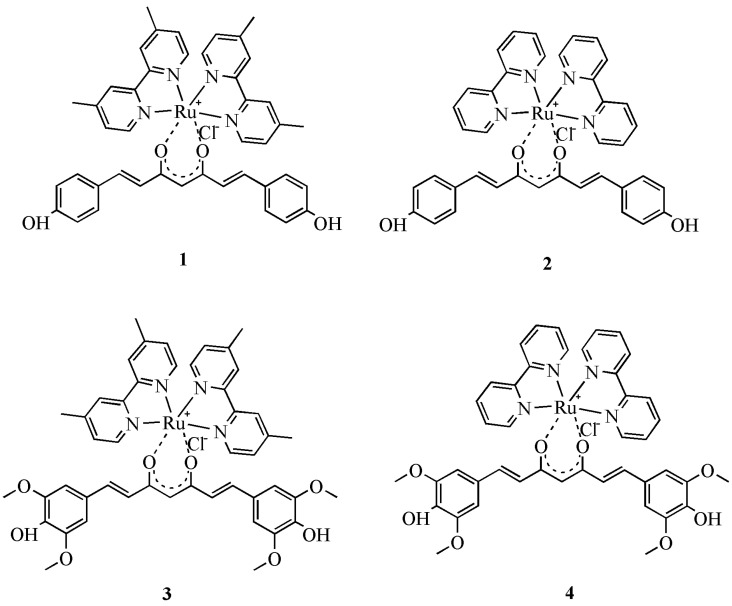
Structures of prepared ruthenium complexes **1**–**4**.

Complexes **1** and **2** contain the bisdemethoxycurcumin (1,7-bis(4-hydroxyphenyl)-hepta-1,6-diene-3,5-dione) ligand **L1**, and complexes **3** and **4** contain the syringaldehyde curcumin (1,7-bis(3,5-dimethoxy-4-hydroxyphenyl)-hepta-1,6-diene-3,5-dione) ligand **L2**. The preparation of complexes **1**–**4** was done following a general procedure by reacting the intermediate ruthenium complexes Ru(dmbpy)_2_Cl_2_ and Ru(bpy)_2_Cl_2_, respectively, with the corresponding curcuminoids **L1** and **L2** in a ratio of 1:1. Reactions were carried out for 3 h at reflux. The final products **1**–**4** (Figure 2) obtained as dark brown powders were purified by silica gel chromatography and structurally characterized by ^1^H NMR, ^13^C NMR, IR spectroscopies, elemental analysis and by MS spectrometry. Attempts for X-ray diffraction measurements have been done for complexes **1**–**4**, prepared in powder form. The X-ray diffraction measurements indicated an amorphous form of the samples as there was not seen any diffraction of the samples. The stability of complexes **1**–**4** was monitored by UV-Vis spectroscopy in an interval of 72 h. UV-Vis spectra were measured each 24 h. No significant changes are observed in the absorption maxima (λ_max_) of the four complexes **1**–**4**, therefore there are no doubts concerning their stability in methanol and in a stock solution (ethanol/water).

### 2.2. Biological Attempts-Cytotoxicity

The ligands **L1**, **L2**, and complexes **1**–**4** display a dose-dependent inhibitory capacity against A2780 ovarian cancer cell populations subjected to in vitro treatment. Different IC_50_ values exhibited by the studied compounds are presented in Table 1. In all cases, the toxicity was time-dependent. The cytotoxicity is associated with low IC_50_ concentrations; ligands **L1** and **L2** exhibited superior IC_50_ values after 24-, 48-, or 72-h of exposure (one-way analysis of variances, Bonferroni’s multiple comparison test in the 95% confidence interval) Thus, ligands **L1** and **L2** are less cytotoxic than the Ru(II) complexes or carboplatin, the standard metal-based drug employed in ovarian cancer treatment. The activity of complexes **1**–**4** folded higher than the activity of ligands in each analyzed time point, with special emphasis on complex **2**, the most cytotoxic.

After 24-h, the IC_50_ values were relatively high for all complexes, above 100 µM, with only one exception: complex **3**, where the values are comparable with that of NAMI-A [41], RAPTA-C [42], KP1019, and AZIRu [43], intensely studied ruthenium compounds against the A2780 cell line. When the exposure was prolonged, the IC_50_ values dropped significantly, especially for **2** and **3**, which denote a fold higher inhibitory capacity. For all compounds, their best inhibitory capacity was recorded after 72 h; three of them: **1**, **2,** and **3** have IC_50_ values under 5 µM, while **4** has an IC_50_ value of 22.9 µM. After 24 h carboplatin cytotoxicity was comparable with **2**, **3**, and **4** (no significant differences, *p* > 0.05), and slightly better than **1**. After 48- and 72-h exposure**,** superior cytotoxicity of **2** and **3** (one-way analysis of variances, *p* < 0.05) was observed. While the populations treated with complexes **2** and **3** evolved rapidly towards cell death, **1** needed 72 h for its best antitumor outcome, and complex **4** increased its toxicity substantially over time (Table 1).

### 2.3. The Influence of Ruthenium Complexes on Nuclear Factor Kappa-Light-Chain-Enhancer of Activated B Cells (NF-κB Transcription Factor) p65 Subunit

**Figure 3:**
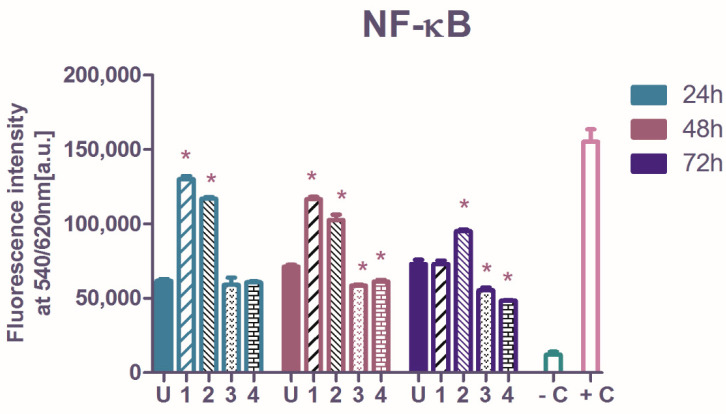
The semiquantitative evaluation of the ruthenium complexes influence on total intracellular NF-*κ*B p65 transcription factor activated in ovarian cancer cells after 24-, 48- or 72-h treatment; the starred columns indicate a significant increase or decrease versus the untreated cells’ NF-*κ*B level. In each interval, the symbol U represents the untreated cells, while **1**–**4** represents the treated cells. The negative control (**−C**) was the fluorescence intensity developed by the cell lysis solution, while the positive control (**+C**) was a reference protein derived from stimulated cells (as described in Section 3). Starred columns represent the significant changes versus the untreated control.

The NF-*κ*B p65 transcription factor level in A2780 cells was reflected by the intensity of fluorescence emission (Figure 3) and for all measurements made on treated or untreated cells, the values were validated as being in the interval between the negative and positive control provided by the manufacturer of the assay kit (Section 3). The IC_50_ concentrations of **1**–**4** were used in the functional tests. The basal NF-*κ*B p65 activation in the untreated A2780 cells had a slightly increasing tendency in time, however without statistical significance.

Relative to untreated cells, the A2780 tumor cells treated with complexes **1** and **2** showed an increase in NF-*κ*B protein levels, after 24- and 48-h exposures, while after 72-h treatment only **2** amplified NF-*κ*B activation. Instead, **3** and **4** act differently. These complexes diminished NF-*κ*B activation after 48- and 72-h exposures (one-way analysis of variance, Dunnett post-test, *p* < 0.01). The ovarian tumors are characterized by elevated constitutive NF-*κ*B expressions, and the dysregulation of NF-*κ*B causes resistance to chemotherapy [44]. The suppression of activated NF-*κ*B is a suitable target in many studies, however, the evidence showed that an intense inhibition of NF-*κ*B only can result in a minimum effect on the tumor [45].

Complexes **3** and **4** displayed a significant inhibition against NF-*κ*B transcription factor, following 48- or 72-h treatment, and revealed their ability to trigger the A2780 cells death through the transcription factor NF-*κ*B pathway. Contrary, the exposure of tumor cells to **1** and **2**, although highly cytotoxic compounds, lead to NF-*κ*B augmentation, which indicates that their cytotoxicity resides in other mechanisms.

### 2.4. The Modulation of Basic Fibroblast Growth Factor (FGF-2)

**Figure 4:**
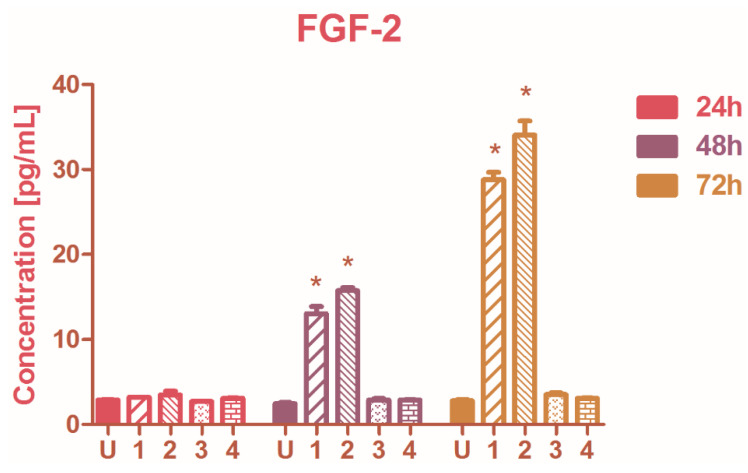
The influence of ruthenium complexes **1**–**4** on FGF-2 secreted by the ovarian cancer cells treated in vitro for 24-, 48-, or 72-h; U represents the untreated control, and the starred columns indicate a significant increase in FGF-2 production in measured up to untreated control cells. Starred columns represent the significant changes versus the untreated control.

The secretion of FGF-2 was modulated by complexes **1** and **2** when the A2780 cells were treated for more than 24 h; the FGF-2 levels increased after the 48 and 72h exposure to **1** and **2** (one-way analysis of variance, *p* < 0.001). No considerable activity was observed in FGF-2 production when treated with compounds **3** or **4** (Figure 4). The FGF-2 level is usually elevated in ovarian tumors and through angiogenesis enhancement, it can promote tumor growth [46]. Conversely, other studies demonstrated that the FGF-2 signaling sensitizes cancer cells to drugs and targeted therapy [47], and in this context, FGF-2 was significantly augmented by **1** and **2**, alongside other mechanisms modulated by Ru(II) complexes that could provide eventually a beneficial antitumor effect as well.

### 2.5. The Influence on Matrix Metalloproteinase-9 (MMP-9) Secretion

**Figure 5:**
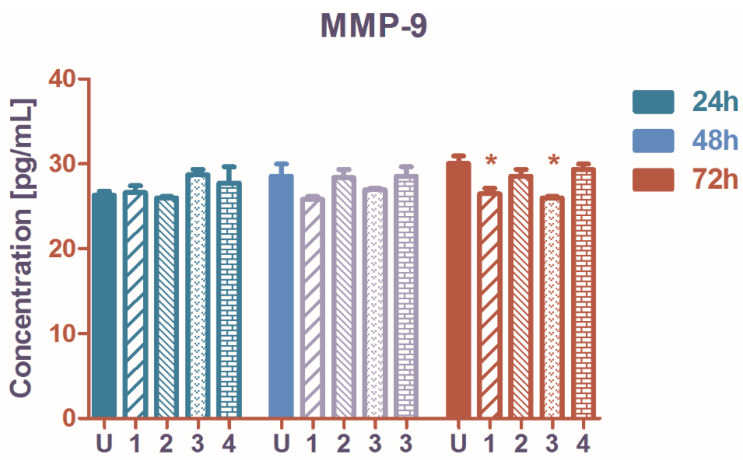
The concentration (pg/mL) of secreted matrix metalloproteinase-9 (MMP-9) in the extracellular medium of A2780 cells treated with ruthenium complexes **1**–**4** in vitro. U represents the untreated cells, **1**,**2**,**3** and **4** the ruthenium complexes; 24-, 48- and 72 h are the exposure times. Starred columns represent the significant changes versus the untreated cells. Starred columns represent the significant changes versus the untreated control.

The A2780 cell population MMP-9 production was only weakly influenced by the treatment with ruthenium complexes **1**–**4**. In comparison with the untreated control, no significant changes were recorded at 24 and at 48 h. At 72 h complexes **1** and **3** reduced the quantity of MMP-9 released into extracellular media (Figure 5). MMP-9 is involved in ovarian cancer progression, metastasis, and platinum-drug resistance [48], and MMP-9 inhibition can improve the standard anticancer treatment efficacy. Acting on the MMP-9 pathway, NF-*κ*B mediated signaling can be initiated for tumor suppression [49].

### 2.6. Interdependence and Connections between the Biologic Parameters

The tumor cell growth inhibitory effect exerted by Ru(II) complexes **1**–**4** was related to NF-*κ*B or FGF-2 pathways and to a certain extent, to MMP-9 modulation. The four parameters are interrelated, and each compound displayed an individual biologic pattern.

**Figure 6:**
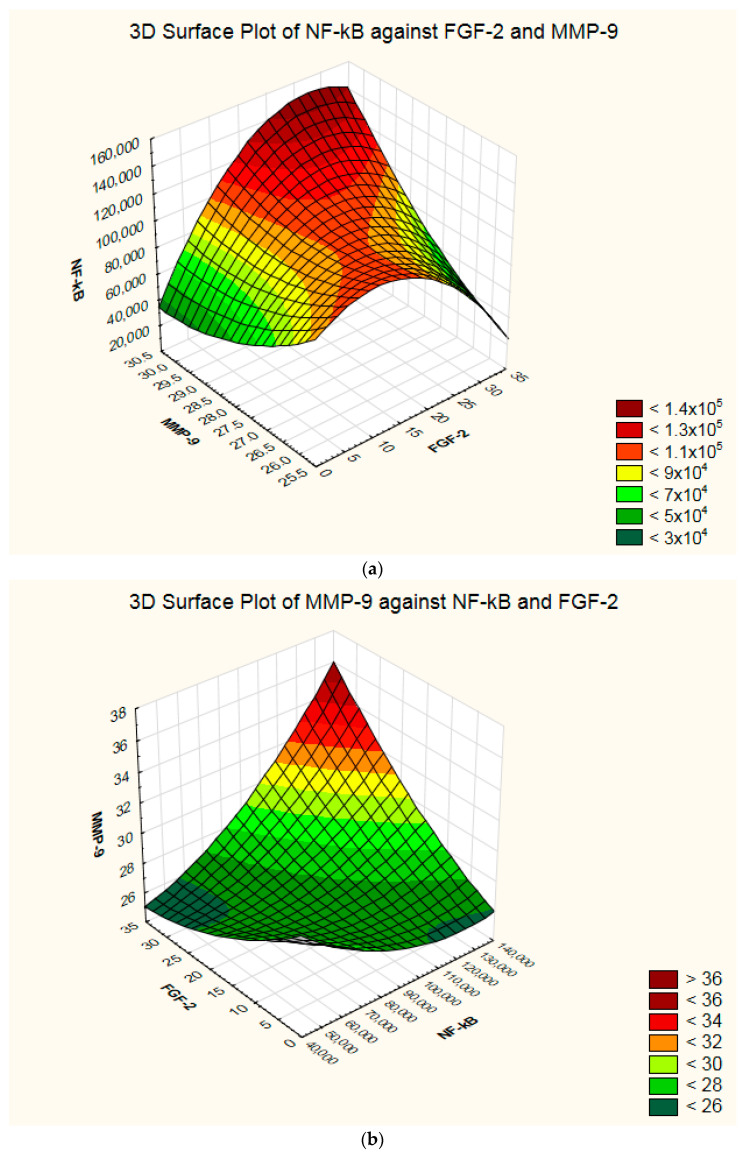

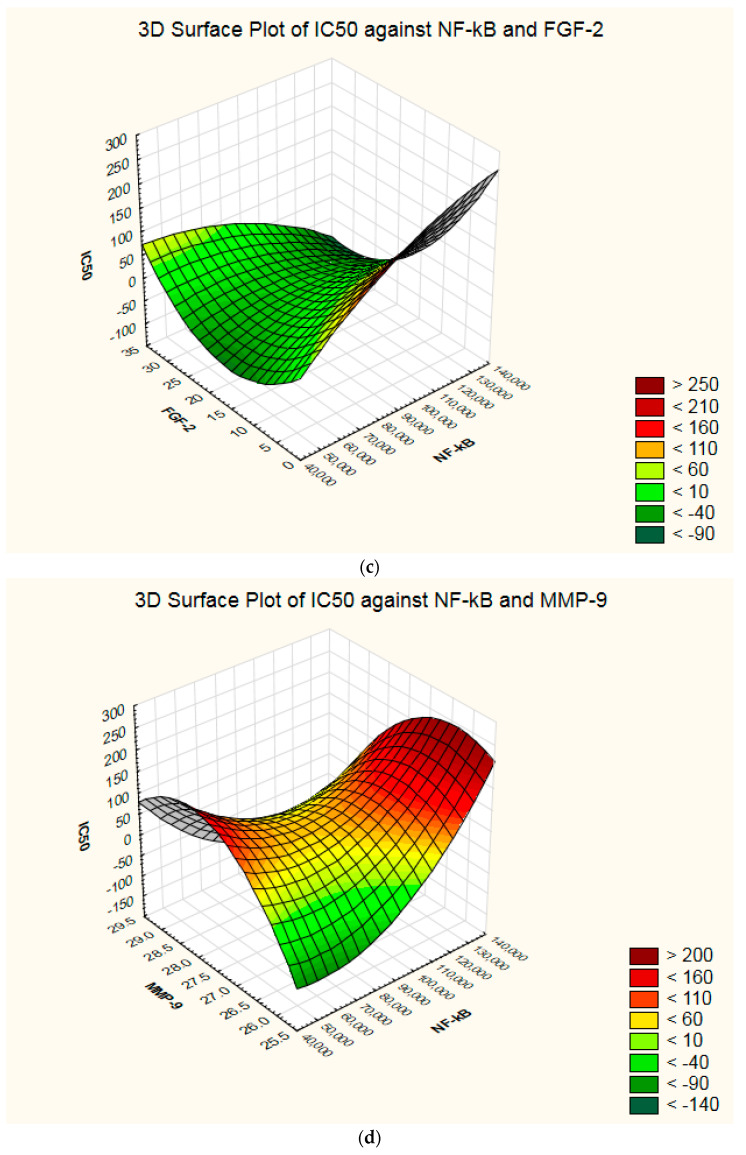
Tridimensional surface plots depicting the covariance between NF-*κ*B, FGF-2, MMP-9 levels, and IC_50_ values in treated A2780 cells. (**a**)—3D Surface Plot of NF-*κ*B against FGF-2 and MMP-9; (**b**)—3D Surface Plot of MMP-9 against NF-*κ*B and FGF-2; (**c**)—3D Surface Plot of IC_50_ against NF-*κ*B and FGF-2; (**d**)—3D Surface Plot of IC_50_ against NF-*κ*B and MMP-9.

The intracellular-activated NF-*κ*B in treated A2780 populations correlates well with the FGF-2 secreted by the cells (nonparametric Spearman correlation, *p*-value 0.047), and there is a significant association between the compounds’ cytotoxicity and FGF-2 (*p*-value 0.029) (Supplementary Figure S1). However, no correlation was observed between the compounds IC_50_ and the total NF-*κ*B level (nonparametric correlation, *p* > 0.05). A 3D representation of the interdependence between the four parameters: IC_50_ concentration, NF-*κ*B, FGF-2, and MMP-9 is illustrated in Figure 6.

The highest NF-κB activation is frequently associated with high MMP-9 and high FGF-2 levels (Figure 6a), complex **2** being characterized by this pattern.

The NF-κB inhibition could be related to the decrease of MMP-9 or FGF-2 (Figure 6b), the best example of a simultaneous drop of NF-κB and MMP-9 being complex **4**.

The high cytotoxicity may be associated with moderated FGF-2 or high NF-κB levels (the case of complex **1**, Figure 6c), while lower cytotoxicity can be associated with a good NF-κB inhibition, such as in the case of complex **3** (Figure 6d).

**Figure 7:**
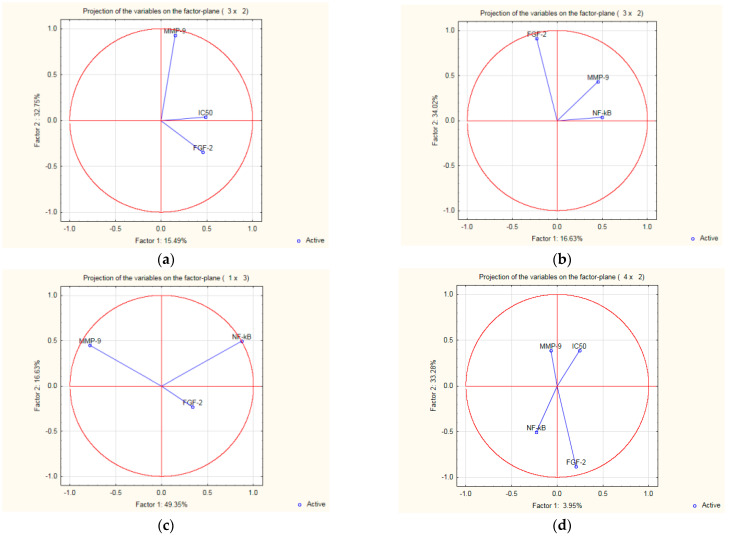
(**a**–**d**). Principal component analysis of the relationship between IC_50_ values, NF-*κ*B transcription factor activation, FGF-2, and MMP-9 levels in A2780 cells treated with **1**, **2**, **3,** and **4**.

The PCA analysis evidenced that among the four variables, IC_50_ (Figure 7a, Tables S1 and S2 in Supplementary material) is the principal component when three variables were considered active (MMP-9 shows as well a good factor). NF-*κ*B (Figure 7b,c) is also a principal component when IC_50_ was not designated as an active component. In the situation when all four parameters were considered active (Figure 7d), IC_50_ exerted the highest influence on the biological outcome of compounds **1**, **2**, **3**, and **4**.

## 3. Materials and Methods

Chemicals used in syntheses (2,2′-dipyridyl, 3,5-dimethoxy-4-hydroxybenzaldehyde, 4,4′-dimethyl-2,2′-dipyridyl, 4-hydroxybenzaldehyde, Acetic acid, Acetone, Acetylacetone, B_2_O_3_, chloroform p.a., DMF, Ethyl acetate p.a., HCl p.a., Hexane, methanol p.a., *n*-butylamine, Ruthenium(III) chloride hydrate (RuCl_3_·xH_2_O), Tri-*n*-butyl borate (98%) were used as purchased and were of reagent grade. Intermediates Ru(bpy)_2_Cl_2_ and Ru(dmbpy)_2_Cl_2_ and the ligands **L1** and **L2**, were prepared according to previously used procedures [50,51].

The ^1^H and ^13^C NMR was measured with a Varian Mercury Plus spectrometer, at a frequency of 400 MHz (for ^1^H-NMR) and 100 MHz (for ^13^C-NMR) from Varian (Agilent Technologies, Santa Clara, California). The used NMR solvent methanol-*d*_4_ (CD_3_OD), was purchased from VWR Eurisotop. Chemical shifts are reported in delta (*δ*) units, given in part per million (ppm) relative to the trimethylsilane (TMS). Coupling constants are reported in Hertz (Hz).

The HR-MS measurements were done in positive mode with a spectrometer LTQ Orbitrap XL.

The infrared spectra (600–4000 cm^−1^) were measured with a Nicolet 6700 FT-IR spectrophotometer.

The UV-Vis spectra (200–800 nm) were recorded with a Genesis 10S UV-Vis spectrophotometer.

The elemental analysis was measured by Flash 2000 CHNS-O Analyzer (Thermo Scientific, Waltham, MA USA).

### 3.1. Synthesis of Ruthenium Complexes

Complex **1**: To a solution of **L1** (0.15 g, 0.47 mmol) in 11 mL mixture of DMF and water (2/1 *v*/*v*: 7.33 mL DMF + 3.66 mL H_2_O), were added Ru(dmbpy)_2_Cl_2_ (0.05 g, 0.09 mmol) and Na_2_CO_3_ (0.05 g, 0.47 mmol). After 3 h of reflux, the solvent was removed in vacuo and the residue was mixed with 15 mL of water. A dark precipitate was formed and then filtered off. The water filtrate was treated with HCl (3 mL, 0.5 M) until the pH was approx. 3. The dark brown precipitate (0.13 g, 35%) was subjected to the silica gel chromatography (CHCl_3_:MeOH, 9:1) to separate the residual co-products. The final product remained on the upper layer of silica gel in the column and therefore was isolated by extraction in MeOH. After removing the solvent, a dark brown powder was obtained as compound **1**. Complex **1**: 0.02 g yield (4%); ^1^H NMR (400 MHz, CD_3_OD), δ (ppm) 2.50 (s, 6H), 2.62 (s, 6H), 5.74 (s, 1H), 6.47 (d, *J* = 15.7, 2H), 6.70 (d, *J* = 8.6, 4H), 6.97 (d, *J* = 15.7, 1H), 7.01 (dd, *J* = 5.9, 1.3, 2H), 7.27 (d, *J* = 8.7, 4H), 7.44 (dd, *J* = 5.8, 1.3, 2H), 7.62 (d, *J* = 5.9, 2H), 8.33 (s, 2H), 8.42 (s, 2H), 8.54 (s, 1H), 8.57 (d, *J* = 5.8, 2H).^13^C NMR (CD_3_OD, 100 MHz) δ (ppm) 19.50 (2C), 19.81 (2C), 54.61 (1C), 115.31 (4C), 123.31 (2C), 125.27 (2C), 125.90 (2C), 126.58 (2C), 127.42 (2C), 128.61 (4C), 136.02 (2C), 146.59 (2C), 148.29 (2C), 149.07 (2C), 151.84 (2C), 157.74 (2C), 158.52 (2C), 159.05 (2C), 168.85 (2C), 178.08 (2C). HR-MS (TOF-ESI+): calcd for C_43_H_39_N_4_O_4_Ru [M + H]^+^: 777.2009, found: 777.2219. IR ν (cm^−1^) 3188, 2924, 1603, 1512, 1417, 1378, 1241, 1167, 1103, 830, 785, 689, 653.Anal. Calc. (%) (C_43_H_39_N_4_O_4_RuCl) C, 63.55; H, 4.83; N, 6.89. Found (%): C, 63.82; H, 4.99; N, 6.75.

Complex **2** [25]: The preparation of complex **2** follows the procedure given for complex **1**. To a solution of **L1** (0.1454 g, 0.47 mmol) in 11 mL of mixture of DMF and water (2/1 *v*/*v*: 7.33 mL DMF + 3.66 mL H_2_O) were added RuCl_2_(bpy)_2_ (0.0455 g, 0.094 mmol), and Na_2_CO_3_ (105.98 g/mol, 0.0498 g, 0.47 mmol). After 3 h of reflux, the solvent was removed in vacuo and the residue was mixed with 18 mL of water. A dark precipitate was formed and then filtered off. The water filtrate was treated with HCl (3 mL, 0.5 M) until the pH was approx. 3. The dark brown precipitate of the crude product (0.2 g, 54.54%) was subjected to the silica gel chromatography (CHCl_3_:MeOH, 9:1) to separate the residual co-products. The final product remained on the upper layer of silica gel in the column and therefore was isolated by extraction in MeOH. After removing the solvent, a dark brown powder was obtained as compound **1**. Complex **2**: 0.04 g yield (12%); ^1^H NMR (400 MHz, CD_3_OD), *δ* (ppm) 5.76 (s, 1H), 6.49 (d, *J* = 15.7, 2H), 6.70 (d, *J* = 8.6, 4H), 6.97 (d, *J* = 15.7, 2H), 7.21 (ddd, *J* = 7.6, 5.8, 1.3, 2H), 7.26 (d, *J* = 8.6, 4H), 7.63 (ddd, *J* = 7.6, 5.6, 1.3, 2H), 7.82–7.86 (m, 4 H), 8.07 (ddd, *J* = 8.2,7.6, 1.5, 2H), 8.51 (dd, *J* = 8.7, 1.2, 2H), 8.59 (dm, *J* = 8.2, *w*_1/2_ = 2.7, 2H), 8.77 (ddd, *J* = 5.6, 1.5, 0.7, 2H). ^13^C NMR (CD_3_OD, 100 MHz) δ (ppm) 102.45 (1C), 115.38 (4C), 122.70 (2C), 122.82 (2C), 125.00 (2C), 125.02 (2C), 125.72 (2C), 127.19 (2C), 128.72 (4C), 134.50 (2C), 136.13 (2C), 136.58 (2C), 149.77 (2C), 152.75 (2C), 158.05 (2C), 158.84 (1 C), 158.87 (1 C), 159.44 (2C), 178.44 (2 C). HR-MS (TOF-ESI+): calcd for C_39_H_31_N_4_O_4_Ru [M + H]^+^: 721.1467, found: 721.1583. IR ν (cm^−1^) 3076, 2926, 1602, 2505, 1462, 1444, 1424, 1262, 1166, 1103, 1021, 977, 833, 762, 729, 659. Anal. Calc. (%) (C_39_H_31_N_4_O_4_RuCl) C, 61.91; H, 4.13; N, 7.40. Found (%): C, 62.28; H, 4.37; N, 7.66.

Complex **3**: To a solution of **L2** (0.2356 g, 0.55 mmol, 5 eq.) RuCl_2_(bpy)_2_ (0.0594 g, 0.110 mmol, 1 eq.), and Na_2_CO_3_ (0.0583 g, 0.55 mmol, 5 eq.) were dissolved in a mixture of DMF and water (13.14 mL, 2/1 *v*/*v*: 8.75 mL DMF + 4.38 mL H_2_O). After 3 h of reflux, the solvent was removed under vacuum and the residue was mixed with 21.5 mL of water. A dark precipitate was formed and then filtered off. The water filtrate was treated with HCl (3.5 mL, 0.5 M) until the pH was approx. 3. The dark brown precipitate (0.5613 g) was subjected to the silica gel chromatography (CHCl_3_:MeOH, 6:1) to filtrate residual coproducts and the final product was extracted to MeOH from the upper layer of silica gel to get dark brown powder. Complex **3**: (0.03 g yield, 27%); ^1^H NMR (400 MHz, CD_3_OD), *δ* (ppm): 2.49 (s, 6H), 2.64 (s, 6H), 3.79 (s, 12H), 5.83 (s, 1H), 6.51 (d, *J* = 15.7, 2H), 6.72 (s, 4H), 7.00 (d, *J* = 15.7, 2H), 7.01 (ddd, *J* = 5.8, 1.9, 0.7), 7.45 (ddd, *J* = 5.8, 1.8, 0.7, 1H), 7.60 (d, *J* = 5.8, 4H), 7.71 (dd, *J* = 5.5, 3.4, 1H), 8.33 (br s, 2H), 8.45 (br s, 2H), 8.59 (d, *J* = 5.8,2H). ^13^C NMR (100 MHz, CD_3_OD) *δ* (ppm): 19.52 (2C), 19.84 (2C), 55.35 (4C), 102.33 (1C), 104.55 (4C), 123.38 (2C), 123.51 (2C), 125.00 (2C), 126.20 (2C), 126.60 (2C), 127.03 (2C), 136.34 (2C), 136.87 (2C), 146.65 (2C), 148.07 (2C), 148.37 (2C), 149.02 (2C), 151.80 (2C), 157.75 (2C), 159.00 (2C), 177.93 (2C).HRMS (TOF-ESI+): calcd for C_47_H_47_N_4_O_8_Ru [M+H]^+^: 897.2437, found: 897.2683. IR ν (cm^−1^) 2935, 1616, 1506, 1455, 1423, 1332, 1092, 824, 731, 673, 661. Anal. Calc. (%) (C_47_H_47_N_4_O_8_RuCl) C, 60.52; H, 5.08; N, 6.01. Found (%): C, 60.93; H, 5.35; N, 6.21.

Complex **4**: The synthesis of complex **4** respects the procedure described for complex **3**. To a solution of **L2** (0.24 g, 0.55 mmol) in DMF and water (13.14 mL, 2/1 *v*/*v*: 8.75 mL DMF + 4.38 mL H_2_O) are added RuCl_2_(bpy)_2_ (0.053 g, 0.11 mmol) and Na_2_CO_3_ (0.06 g, 0.55 mmol). After 3 h of reflux, the solvent was removed under vacuum and the residue was mixed with 21.5 mL of water. A dark precipitate was formed and then filtered off and the filtrate was treated with HCl (3.5 mL, 0.5 M) until the pH was approx. 3. The final product **4** was isolated by silica gel chromatography (CHCl_3_:MeOH, 7:1→ 4:1) as a dark brown powder (0.04 g yield, 44%). Complex **4**: ^1^H NMR (400 MHz, CD_3_OD), *δ* (ppm): 3.81 (s, 12H), 5.870 (s, 1H), 6.56 (d, *J* = 15.7, 2H), 6.74(s, 4H), 7.01 (d, *J* = 15.7, 2H) 7.22 (m, 2H), 7.65 (ddd, *J* = 7.2, 5.6, 1.1, 2H), 7.83–7.87 (m, 4H), 8.09 (m, 2H), 8.52 (d, *J* = 8.8, 2H), 8.61 (d, *J* = 8.2, 2H), 8.78 (d, *J* = 5.1, 2H). ^13^C NMR (CD_3_OD, 100 MHz) *δ* (ppm): 54.55 (4C), 101.63 (1C), 103.84 (4C), 121.96 (2C), 122.08 (2C), 124.30 (2C), 124.94 (2C), 125.15 (2C), 126.09 (2C), 133.77 (2C), 135.37 (2C), 136.07 (2C), 136.18 (2C), 147.24 (4C), 148.90 (2C), 151.90 (2C), 157.24 (2C), 158.58 (2C), 177.50 (2C). HRMS (TOF-ESI+): calcd for C_43_H_39_N_4_O_8_Ru [M+H]^+^: 841.1889, found: 841.2006. IR ν (cm^−1^) 3364, 2935, 1720, 1720, 1601, 1509, 1461, 1423, 1331, 1248, 1267, 1216, 1155, 1112, 1021, 914, 828, 766, 730, 659.Anal. Calc. (%) (C_43_H_39_N_4_O_8_RuCl) C, 58.91; H, 4.48; N, 6.39. Found (%): C, 58.62; H, 4.73; N, 6.81.

### 3.2. Biological Testing—Cell Growth Inhibition

The A2780 human ovarian adenocarcinoma cells were acquired from the European Collection of Authenticated Cell Cultures (ECACC, through Sigma Aldrich, St. Louis, MO, USA). The cells were grown in RPMI-1640 cell culture media supplemented with 10% fetal bovine serum, from Sigma Aldrich Company, St. Louis, MO, USA. The in vitro testing was performed in a cell culture laboratory fully equipped with Revco RGT-5000T-9-VBC CO2 Incubator (from Thermo Electron Corporation, Asheville, NC, USA), 32R centrifuge from Hettich Lab Technology, Tuttlingen, Germany; Streamline Class II Biological Safety Cabinet from Esco, Changi, Singapore; Observer D.1 inverted phase fluorescence microscope from Carl Zeiss, Jena, Germany; Synergy 2.0 microplate reader from BioTek Company, Winooski, VT, USA; Tecan Sunrise ELISA plate reader from Tecan Group, Männedorf, Switzerland); Arctiko Uluf-750 vertical −80 °C ultrafreezer from Esbjerg, Denmark; Automatic cell counter Eve from NanoEnTek, Seoul, Korea and PSU-10i orbital shaker from BioSan, Riga, Latvia.

The curcuminoid ligands **L1** (MW 308.328) and **L2** (MW 428.432) and the four ruthenium complexes: **1** (MW 812.329), **2** (MW 756.221), **3** (MW 932.433), and **4** (MW 876.325) were weighted (Extend ED124S analytical balance from Sartorius, Göttingen, Germany) and dissolved in absolute ethanol (from Honeywell Riedel-de-Haen A.G., Seelze, Germany) to obtain a 5 mM stock solution from each complex. Serial dilutions were prepared using RPMI-1640 cell culture media, to obtain nine successive concentrations. As a reference, the standard antitumor drug carboplatin was used (from Teva Pharmaceuticals SRL, Bucharest, Romania).

The MTT (3-(4,5-dimethylthiazol-2-yl)-2,5-diphenyltetrazolium bromide, from Sigma Aldrich) cytotoxicity test was completed for each compound after the A2780 tumor cells exposure to ruthenium complexes for 24, 48 and 72 h, respectively. To perform the cytotoxicity testing, 96-well microplates (Nunclon Delta, from Nalgene Nunc through Thermo Scientific Company, Waltham, MA, USA) were seeded with 10^4^ cells in 190 µL cell culture media and incubated for 24 h preceding the testing. The cells were treated in triplicates (three wells for each concentration) with 10 µL of **L1**, **L2**, **1**, **2**, **3**, **4**, and carboplatin; the final concentrations in the cell culture media were 250, 125, 62.50, 31.25 µM, 15.63, 7.81, 3.91, 1.95, and 0.98 µM. As a reference, untreated cells were used, and as a negative control, wells loaded with culture media only, without cells. On each plate, 9 color controls were dispensed, consisting of cell culture media with 10 µL of the compound from each concentration. Separate 96-well plates were prepared for the 24-, 48- and 72-h experiments. All experiments were repeated three times.

After 24-, 48- or 72 h of the treatment, the cell culture media was removed from the wells. A total of 100 µL 1 mg/mL MTT solution was dispensed in each well and incubated for one hour. Then, the plates were emptied, and 150 µL DMSO was pipetted in each well, and after a short shake, the plates were read in absorbance at 570 nm using the microplate reader. The half-maximal inhibitory concentrations (IC_50_) for each compound were computed, based on the sigmoidal dose-response curves generated by the biostatistics software.

ELISA testing was performed to measure the level of relevant soluble proteins and transcription factors. The cells were loaded on 12-well assay plates (Nunclon Delta, from Nalgene Nunc), in 1425 µL media, at a concentration of 10^5^ cells/mL. Three plates were prepared for each treatment period (24, 48, and 72 h) for all compounds, with 10 wells filled. The next day, after the cells adhered to the surface of the wells, the plates were treated with complexes **1**–**4**; two different wells were treated for each sample with a 75 µL solution of **1**, **2**, **3**, or **4**. In all cases, the final concentration of the ruthenium complex in the cell suspension was identical with their IC_50_ concentration, mentioned in Table 1 for each treatment interval.

Three plates were processed after 24 h; to determine the soluble FGF-2 and MMP-9 levels. The supernates were collected, centrifuged at 4000 RPM aliquoted, and stored at −80 °C.

Subsequently, to obtain the cell lysates for the intracellular *NF-κ*B p65 subunit, the plates were washed gently with warm PBS; the cells were harvested from each well and counted using the automatic cell counter. The cell pellets were treated with a lysis solution provided by the ELISA kit (details below in the ELISA method description), collected, and kept in the ultrafreezer at −80 °C.

The procedures were repeated after 48 and 72 h of treatment, and when all samples were ready, the samples’ protein concentration was normalized according to the number of cells comprised in each well and subjected to ELISA testing.

### 3.3. Soluble Basic Fibroblast Growth Factor (FGF-2) Production

The level of FGF-2 secreted by the cells was measured using the Human FGF basic immunoassay Quantikine ELISA from R&D Systems (Minneapolis, MN, USA) following the manufacturer’s protocol. The assay plate coated with a monoclonal antibody specific for human FGF basic was loaded with 100 μL of Assay Diluent RD1-43, then 100 µL of FGF-2 standard solutions (9 serial concentrations between 1.25 and 320 pg/mL), calibrator diluent as negative control and supernate samples were added, all in duplicates. The plate was covered and incubated for 2 h at room temperature. All strips were exposed to automatic washing; four wash cycles with the wash solution provided by the manufacturer. Afterward, 200 μL of anti-FGF basic human monoclonal antibody conjugated to horseradish peroxidase (HRP) was added to each well and incubated for another 2 h at room temperature. The wash cycles were repeated, and after that, the wells were filled with 200 μL of substrate solution (1:1 mix of stabilized tetramethylbenzidine and hydrogen peroxide solutions) and incubated for 30 min in the dark. The stop solution was added, and the plate was measured in colorimetry at 540/450 nm, using the ELISA reader. Based on the optical density of the samples, individual concentrations were computed.

### 3.4. Matrix Metalloproteinase-9 (MMP-9)

The test was performed with the Human MMP-9 ELISA kit from Invitrogen, BenderMedSystems GmbH, Vienna, Austria, as described earlier [52]. Briefly, the 96-well assay plate coated with anti-MMP-9 antibody was washed and then loaded with 100 µL standards (9 serial concentrations in the range of 0.017–7500.00 ng/mL, in duplicates), 100 µL of assay buffer as blank, 100 µL of samples, in duplicates. A total of 50 µL biotin-conjugated anti-MMP-9 antibody solution was dispensed in each well, and the plate was incubated with shaking for 2 h. The plate was washed four times, 100 µL of Streptavidin-HRP solution was added to each well, and the plate was incubated for 1 h. After another wash cycle, the wells were loaded with 100 µL TMB substrate and after 15 min, a stop solution was added to the plate, and the samples were read with the ELISA reader, at 540/450 nm. The standard curve was created, and individual concentrations were plotted by the Magellan software of the ELISA Equipment.

### 3.5. The Intracellular Nuclear Factor Kappa-Light-Chain-Enhancer of Activated B Cells (NF-κB)

The semi-quantitative measurement of the NF-*κ*B transcription factor p65 subunit was determined with the PhosphoTracer NF-*κ*B p65 ELISA Kit from Abcam, Cambridge, UK. The samples (treated cell pellets) processed as described above, have been subjected to lysis, using the lysis buffer: enhancer solution 1:5 mix provided by the ELISA kit, on an orbital shaker platform (100 RPM) at room temp for 10 min. The 8-well strips of the ELISA microplate were loaded with 50 µL cell lysates, in duplicate, with lysis mix as a negative control, and positive controls provided by the kit, consisting of lysed activated cells, reconstituted in 250 μL purified H_2_O. A total of 50 μL/well of 1:1 Capture Antibody: Detection Antibody Mix was added to each well, and the plate was covered with foil and incubated at room temperature for 1 h while shaking (100 RPM). The wells were washed three times with the wash solution included in the kit, and in the drained wells 100 μL substrate mix was added. The plates were incubated at dark for 10 min, 10 μL of stop solution was dispensed in each well, and the plates were read in fluorescence at 540/25 nm excitation and 620/40 nm emission wavelengths, with high sensitivity signal capture. The fluorescence intensity of the positive control was 155.179 ± 8.426 [a.u], and that of the negative control was 12.003 ± 2.026. The measurements corresponding to all samples were in between these two assessments. For all exposure intervals (24-, 48-, and 72-h, respectively), the untreated cells’ fluorescence intensity was used as a reference value.

The data were analyzed using the GraphPad Prism5 (from GraphPad Software, La Jolla, CA, USA) and Statistica 12.5 (StatSoft Inc., Tulsa, OK, USA) software.

## 4. Conclusions

Curcumin and its derivates are capable of influencing carboplatin resistance, and Ru(II) as central metal is able to inhibit tumor growth by acting through a variety of cell death mechanisms [53]. The synthesized compounds **1**–**4**, where curcuminoid ligands **L1** and **L2** coordinate the metal center ruthenium, achieved a good antitumor response. The highest cytotoxicity belongs to **2**, while **3** is the best to target the cell death mechanisms through NF-*κ*B or MMP-9 signaling. The activity of the synthesized Ru(II) complexes **1**–**4** depends on the length of treatment and the late modulation of MMP-9 and FGF-2 confirms also this tendency. However, a constant decrease of NF-*κ*B after the treatment with complexes **3** and **4**, proves the compounds’ modulator capacity in all phases of the exposure.

The antitumor potential of curcumin can be improved by incorporation into nanostructures [54]; moreover, the efficacy of the Ru(II) complexes against A2780 cells increased significantly when embedded in antitumor-targeted nanoparticles [55], therefore this could be the next step in the development of such compounds to obtain potential prodrugs.

## Figures and Tables

**Table 1:** Half maximal inhibitory concentrations (best-fit IC_50_ values, µM) of ruthenium complexes **1**–**4**, a mathematical parameter reflecting their cytotoxicity against A2780 ovarian cancer cells in vitro (SEM—standard error of the mean, resulted from three independent experiments).

IC_50_Concentration (µM)	L1		L2		Complex 1		Complex 2		Complex 3		Complex 4		Carboplatin	
Duration of Treatment	IC_50_	SEM	IC_50_	SEM	IC_50_	SEM	IC_50_	SEM	IC_50_	SEM	IC_50_	SEM	IC_50_	SEM
24 h	**210.29**	8.12	**226.91**	7.61	**196.72**	8.49	**143.55**	6.948	**82.81**	13.10	**113.36**	4.69	**103.78**	6.51
48 h	**130.63**	3.45	**184.12**	6.50	**48.33**	4.06	**3.12**	0.050	**5.29**	0.41	**78.87**	2.99	**37.04**	2.24
72 h	**107.85**	3.34	**126.96**	14.76	**1.32**	0.11	**0.50**	0.064	**3.35**	0.05	**22.92**	1.26	**9.52**	0.13

## Data Availability

Not applicable.

## Supplementary Materials

The following supporting information can be downloaded at: https://www.mdpi.com/article/10.3390/molecules27144565/s1. Table S1: Eigenvalues provided by the principal component analysis, based on active variables NF-*κ*B, FGF-2, and MMP-9, in correlation with IC_50_ values reflecting cytotoxicity; Table S2: Correlation matrix of factor coordinates generated by PCA, corresponding to the variations of IC_50_, NF-*κ*B, FGF-2 and MMP-9 in A2780 cells following the time-dependent treatment with the series of novel synthesized ruthenium complexes **1**–**4**; Figure S1: The intracellular activated NF-*κ*B p65 in treated A2780 cells (a) correlates well with FGF-2 secreted by the cells (nonparametric Spearman correlation coefficient r = 0.4665, *p*-value 0.0398), and (b) increases inversely proportional to MMP-9 (r = −0.4592, *p*-value 0.0425). As well, it is a significant negative correlation between (c) the compounds cytotoxicity and FGF-2 (r = −0.5604, *p*-value 0.0290).

## Sample Availability

Samples of the compounds are available from the authors.
