# Supplementary material for: Antiproliferative Ruthenium Complexes Containing Curcuminoid Ligands Tested In Vitro on Human Ovarian Tumor Cell Line A2780, towards Their Capability to Modulate the NF-κBTranscription Factor, FGF-2 Growth Factor, and MMP-9 Pathway
Source: Molecules. 2022 Jul 18;27(14):4565. doi: 10.3390/molecules27144565 (PMC9322753; doi:10.3390/molecules27144565)

Supplementary material

Antiproliferative ruthenium complexes containing curcuminoid ligands tested *in vitro* on human ovarian tumor cell line A2780, towards their capability to modulate the NF-κB transcription factor, FGF-2 growth factor and MMP-9 pathway

Janka Leskovská [1*], Natalia Miklášová[1*], Paul-Milan Kubelac[2,3], Patriciu Achimas-Cadariu[2,4], Jindra Valentová[1], Mário Markuliak[1], Eva Fischer-Fodor[2]

[1] Department of Chemical Theory of Drugs, Faculty of Pharmacy, Comenius University in Bratislava, Kalinčiakova 8, 83104 Bratislava, Slovakialeskovska5@uniba.sk (J.L.); miklasova@fpharm.uniba.sk (N.M.), valentova@fpharm.uniba.sk (J.V); markuliak@fpharm.uniba.sk (M.M.)

[2] Institute of Oncology "Prof.Dr.I.Chiricuta" Cluj-Napoca, RO-400015, Romania; fischer.eva@iocn.ro (E.F-F.), paulkubelac@yahoo.com (P.-M.K.), patrick.achimas@hotmail.com (P.A.-C.)

[3] Department of Oncology, "Iuliu Hatieganu" University of Medicine and Pharmacy, RO-400012, Cluj-Napoca, Romania

[4] Department of Oncological Surgery and Gynecological Oncology, "Iuliu Hatieganu" University of Medicine and Pharmacy, RO-400012, Cluj-Napoca, Romania.

* Correspondence: leskovska5@uniba.sk (J.L.); miklasova@fpharm.uniba.sk (N.M.); Tel.: +421-250-117-322 (J.L.); +421-250-117-327 (N.M.)

NMR, HRMS and IR spectra of free ligands and ruthenium complexes 1,2,3 and 4

NMR Spectrum of **BDMC**

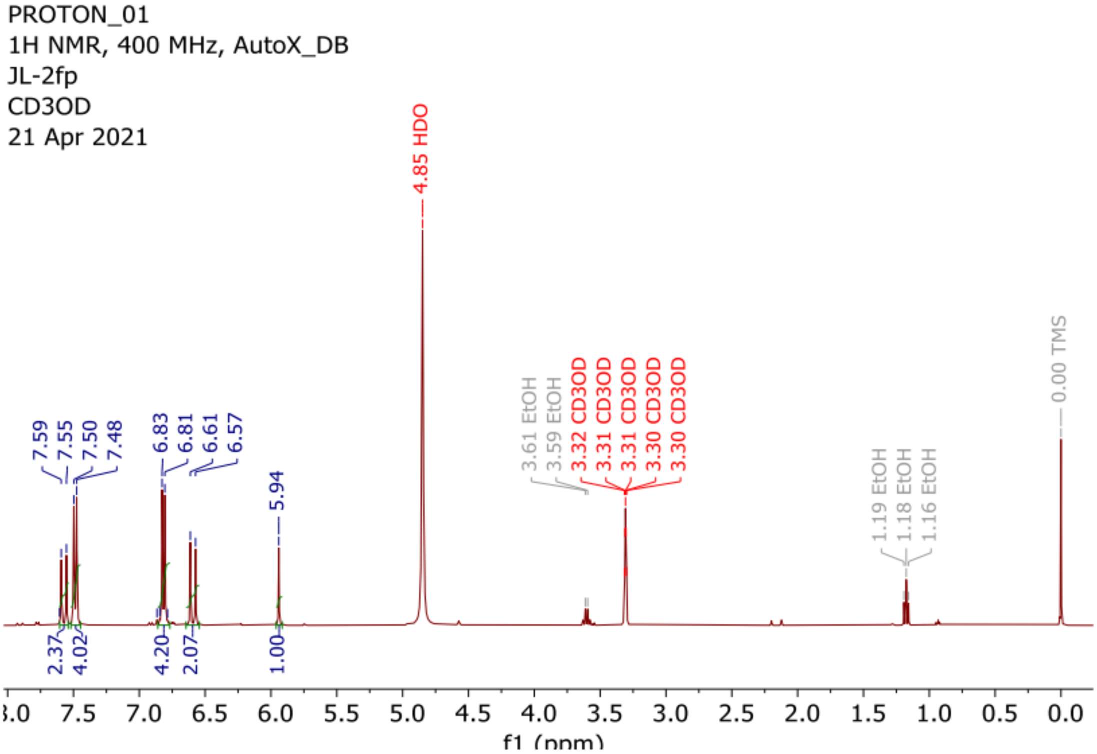

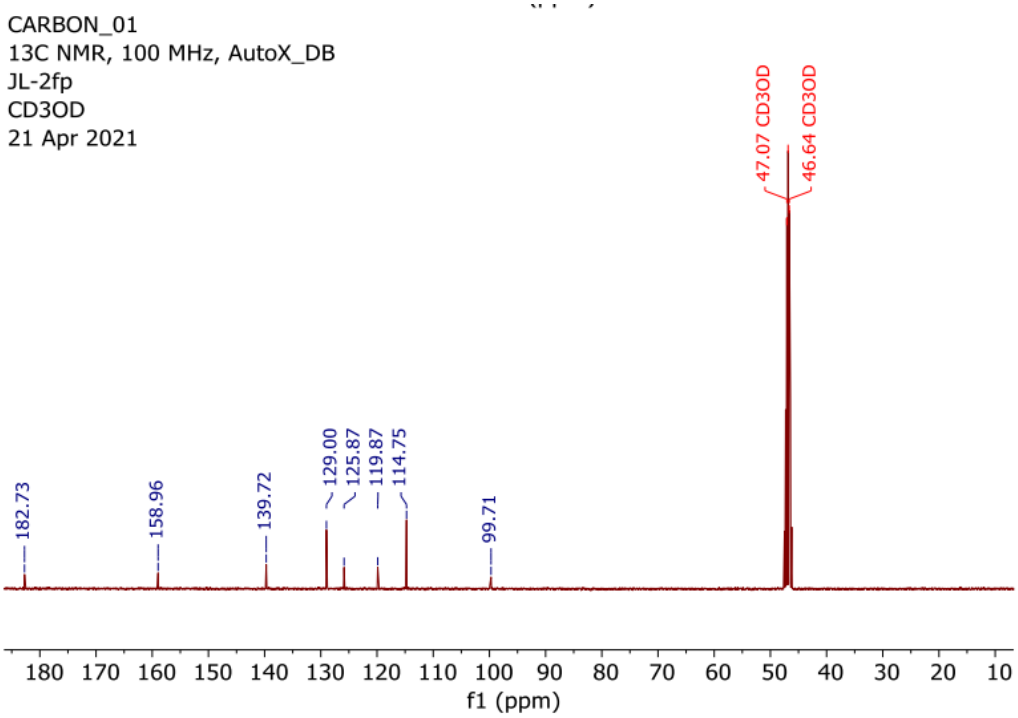

NMR Spectrum of **Syringaldehyde curcumin**

PROTON_01
1H NMR, 400 MHz, AutoX_DB
NM-167
CDCl3
10 Jun 2019

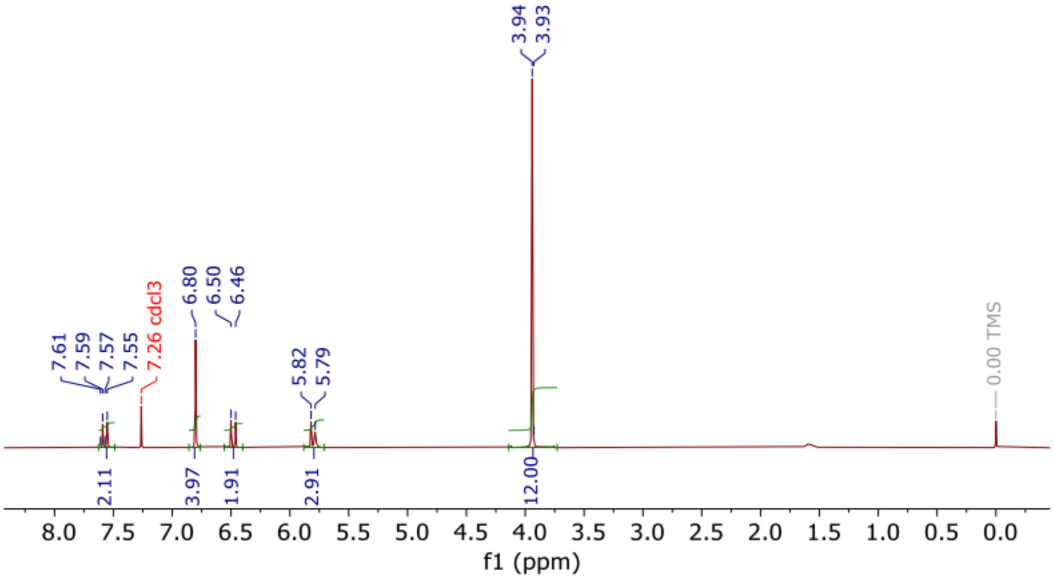

CARBON_01
13C NMR, 100 MHz, AutoX_DB
NM-167
CDCl3
11 Jun 2019

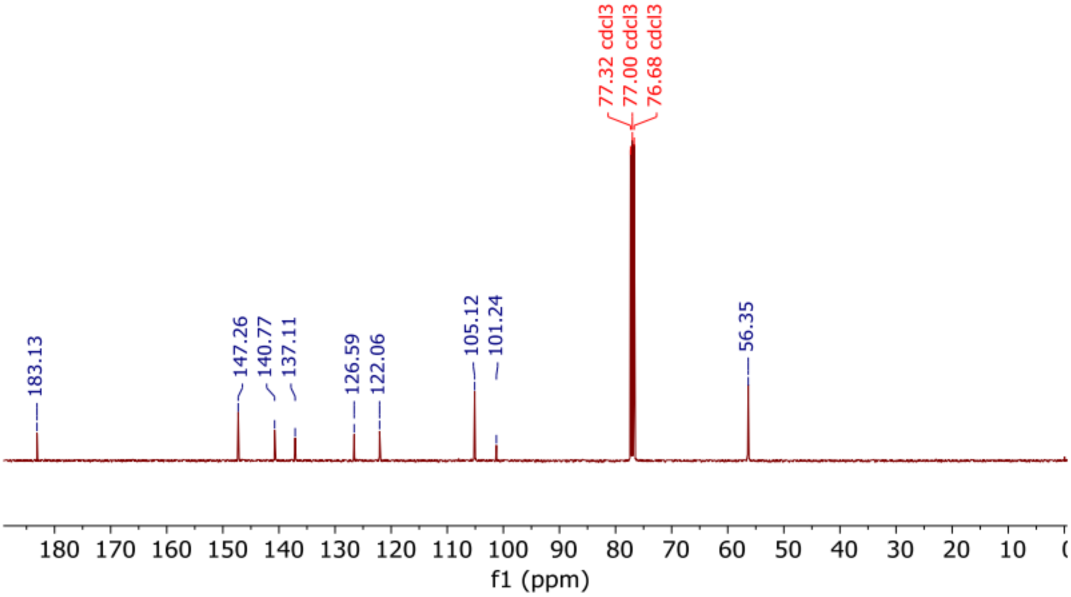

NMR Spectrum of complex 1

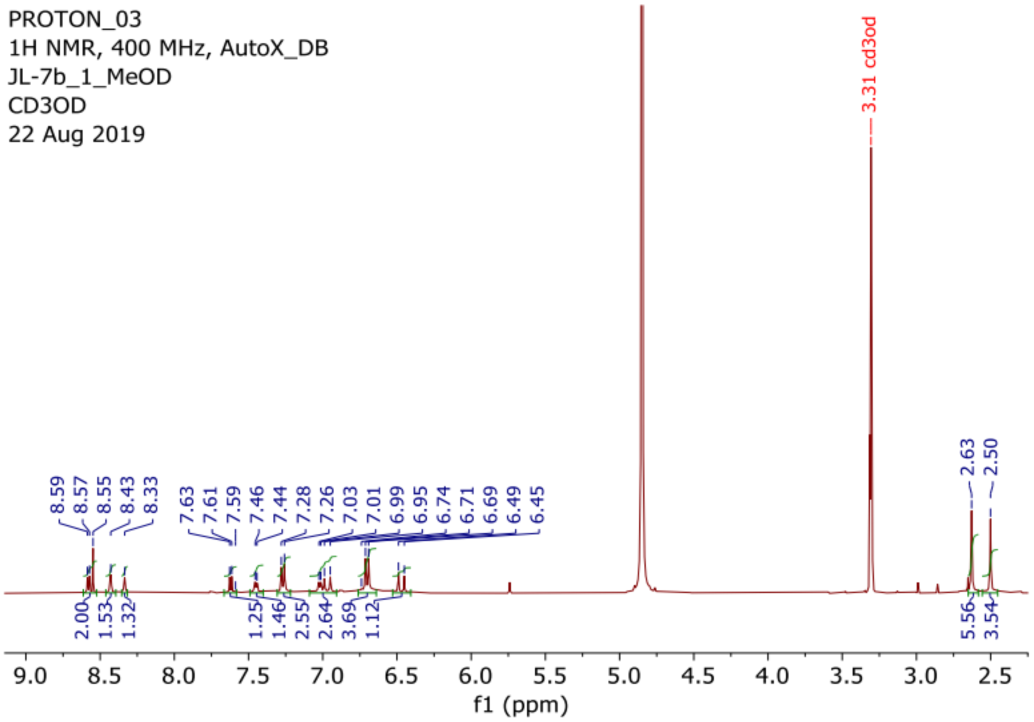

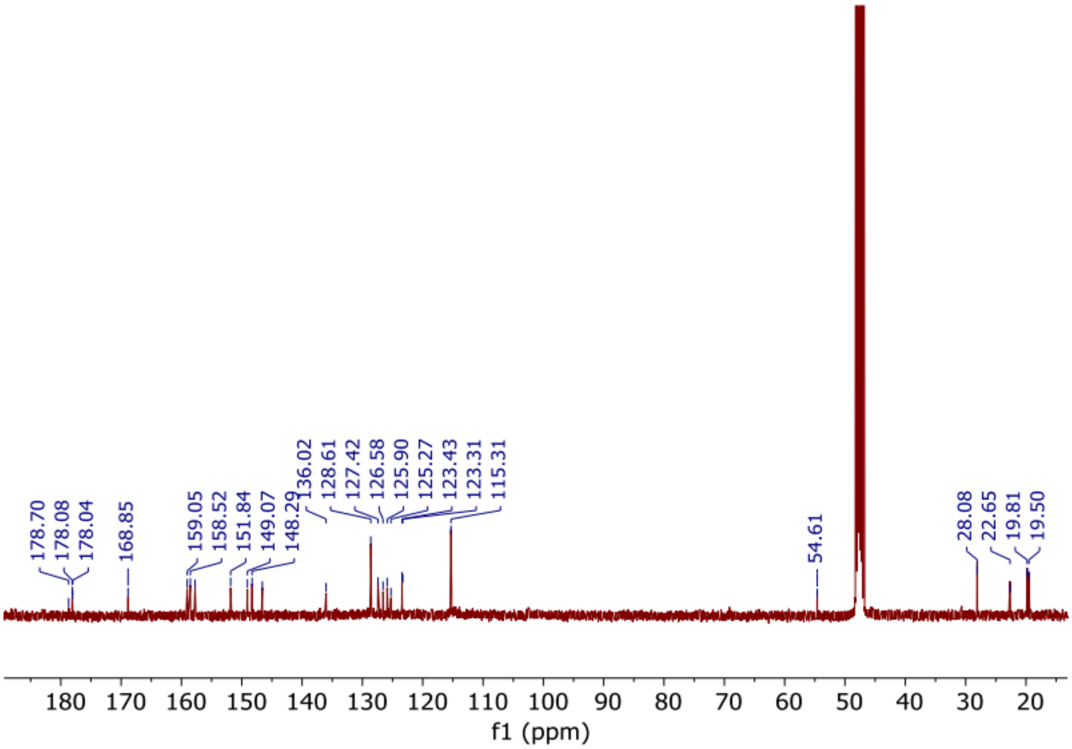

2D NMR measurements confirming the structure of complex **1**

DQCOSY

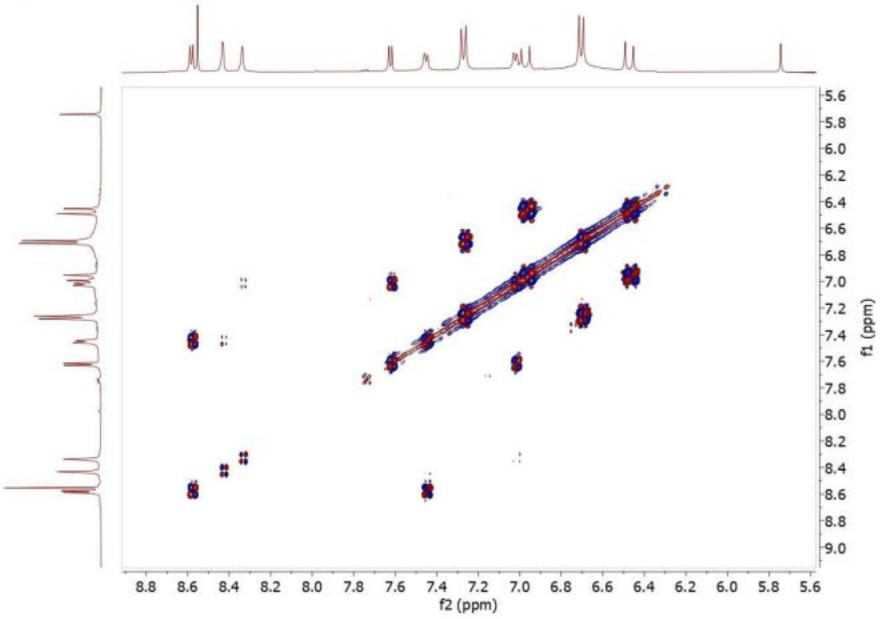

HSQC

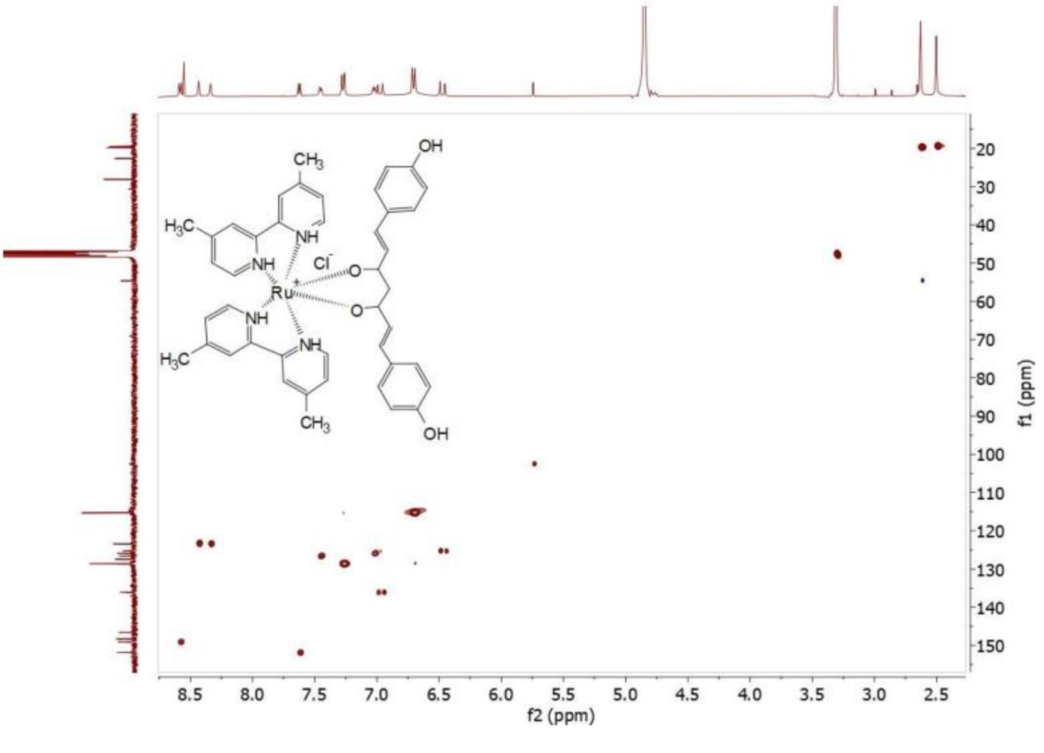

HMBC

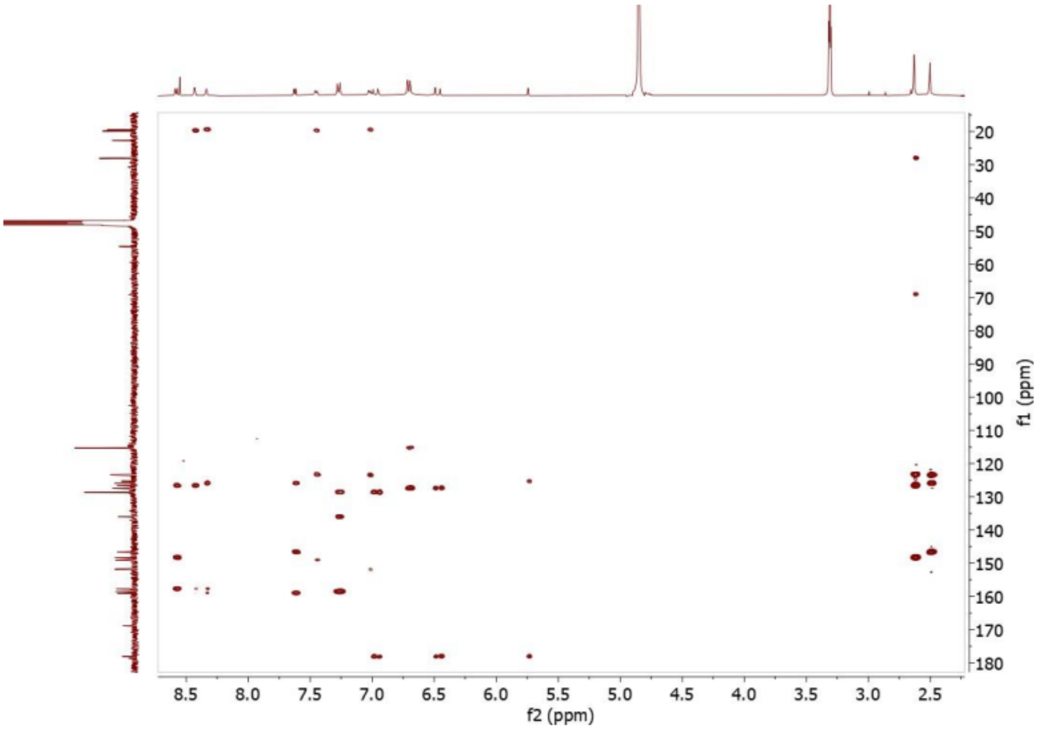

NMR of Complex **2**

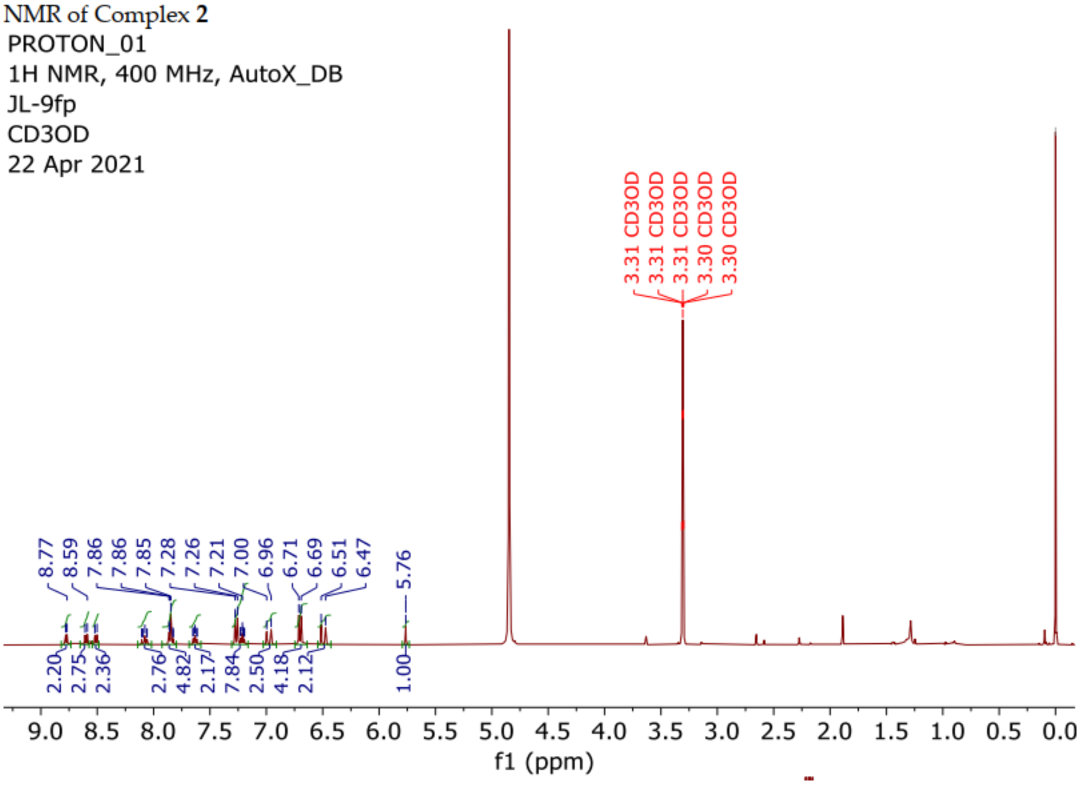

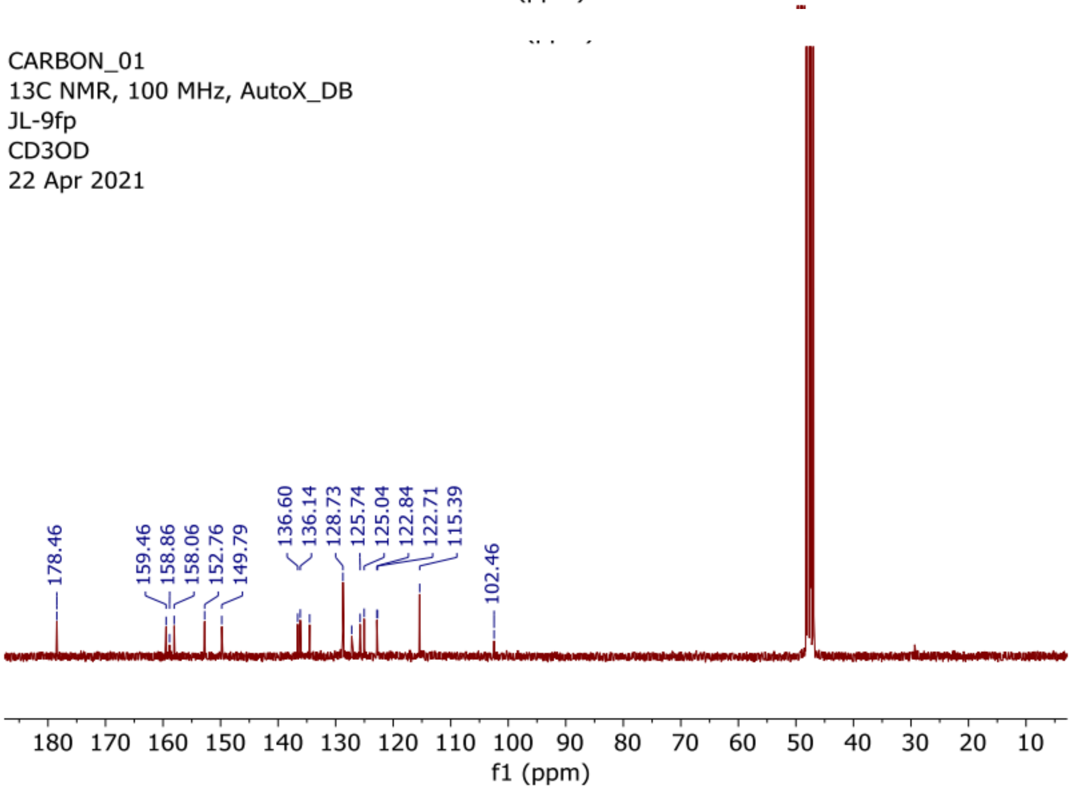

NMR of Complex **3**

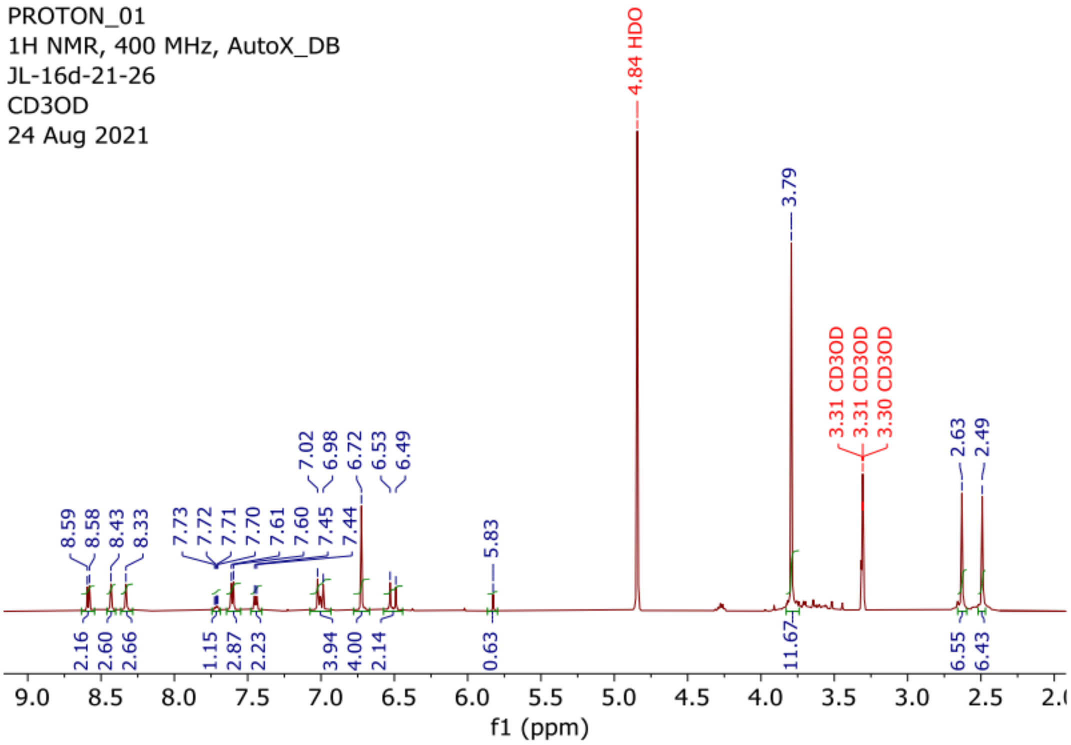

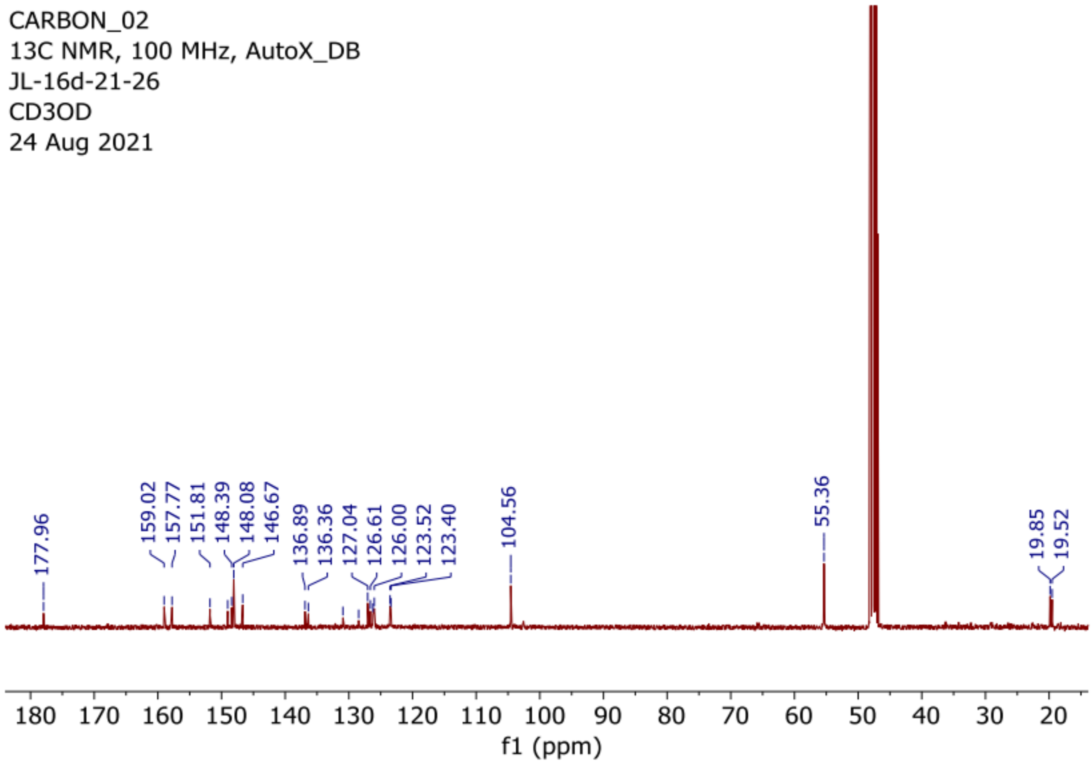

NMR of Complex 4

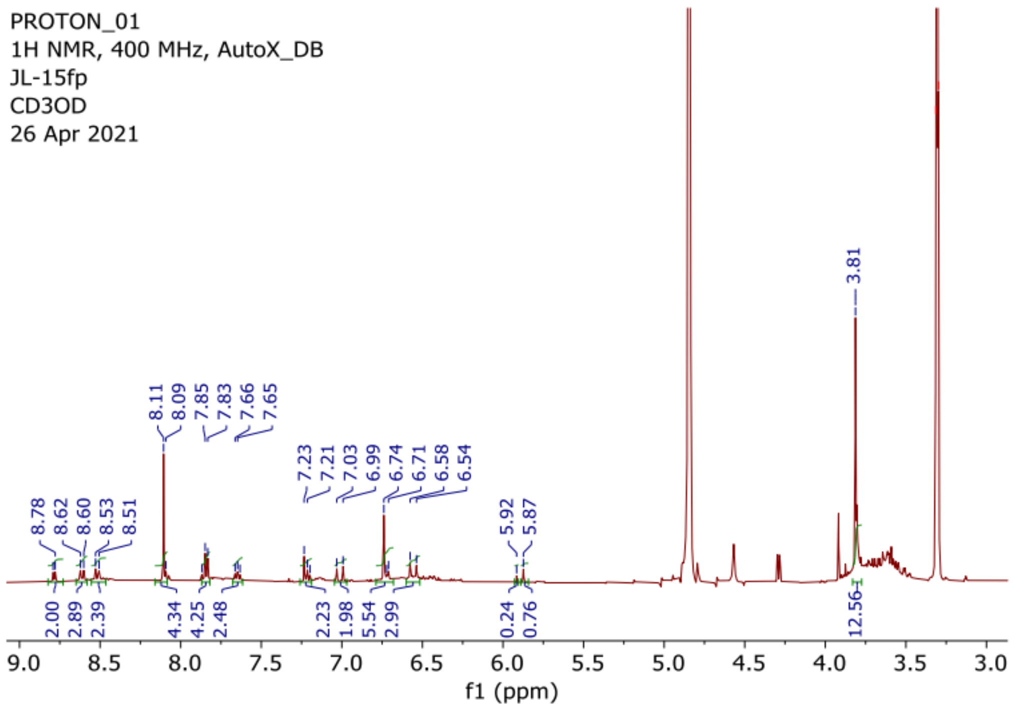

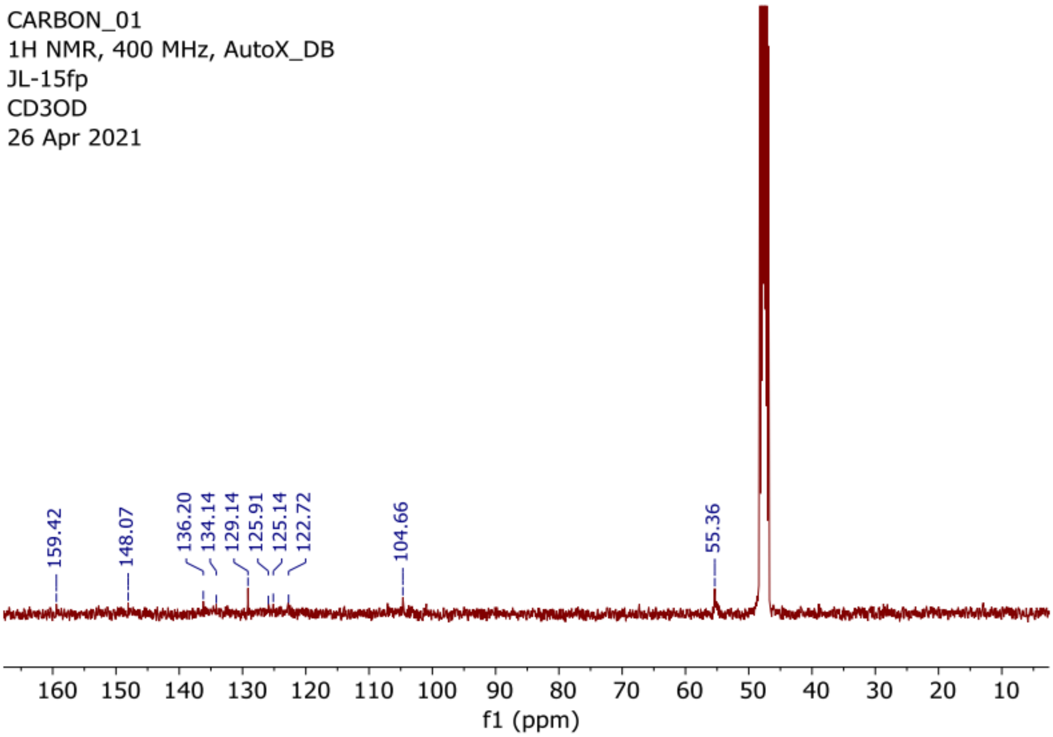

HR-MS data

Complex 1

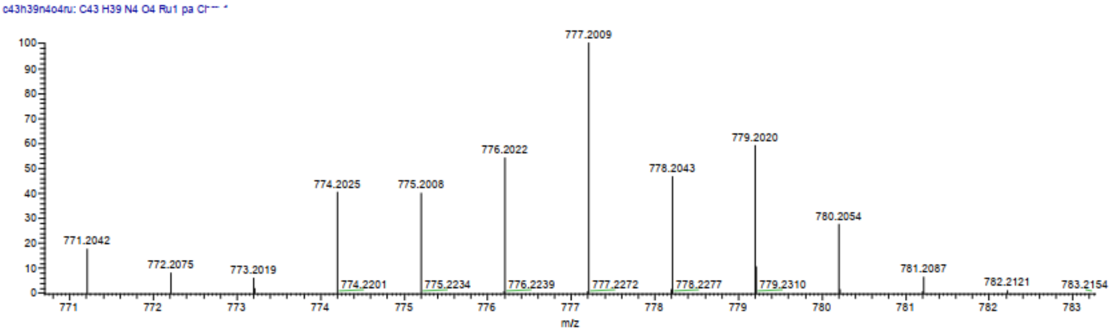

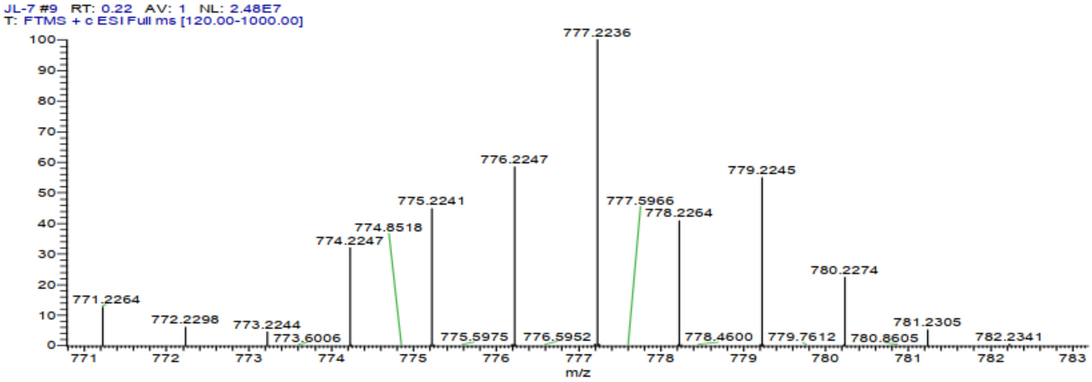

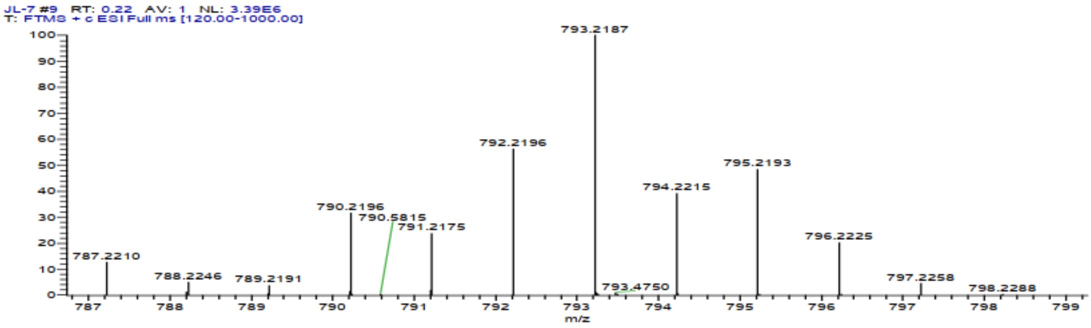

Complex 2

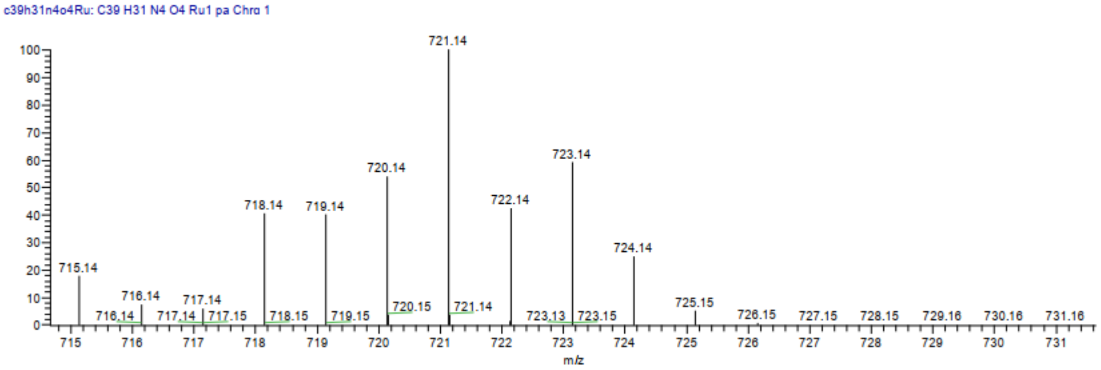
c39h31n4o4Ru: C39 H31 N4 O4 Ru1 pa Chrg 1
721.14
723.14
720.14
722.14
718.14
719.14
724.14
715.14
716.14
717.14
725.15
m/z

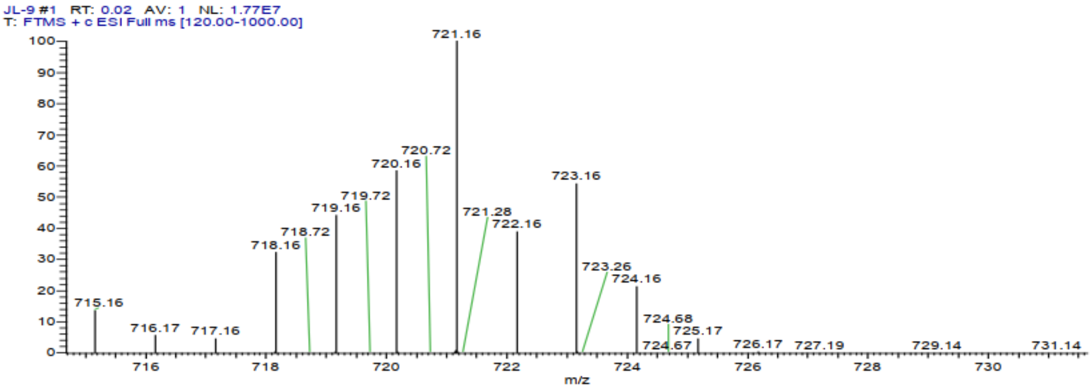
JL-9 #1 RT: 0.02 AV: 1 NL: 1.77E7
T: FTMS + c ESI Full ms [120.00-1000.00]
721.16
720.72
720.16
723.16
719.72
719.16
721.28
722.16
718.72
718.16
723.26
724.16
715.16
724.68
725.17
716.17
717.16
m/z

Complex 3

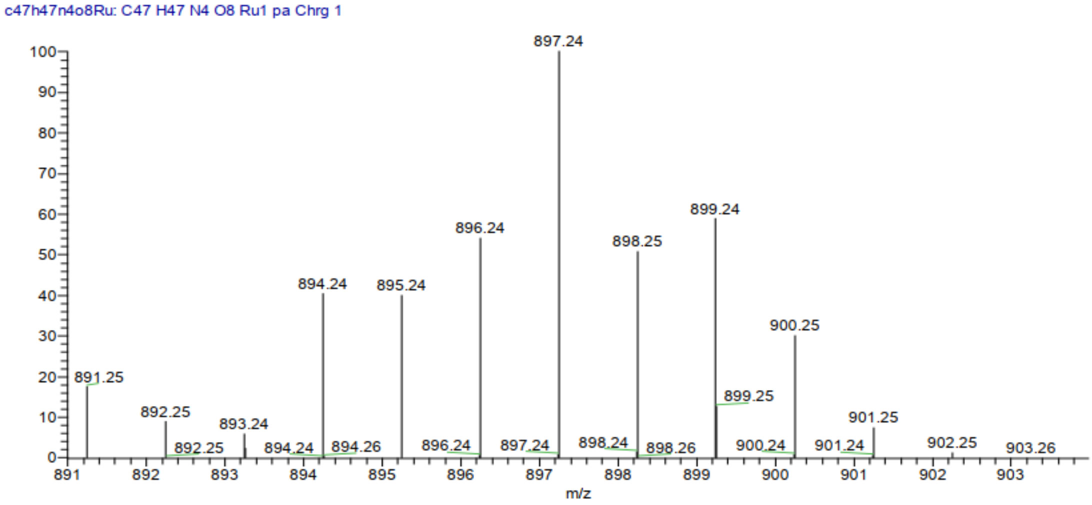
c47h47n4o8Ru: C47 H47 N4 O8 Ru1 pa Chrg 1
897.24
899.24
896.24
898.25
894.24
895.24
900.25
891.25
899.25
892.25
893.24
901.25
m/z

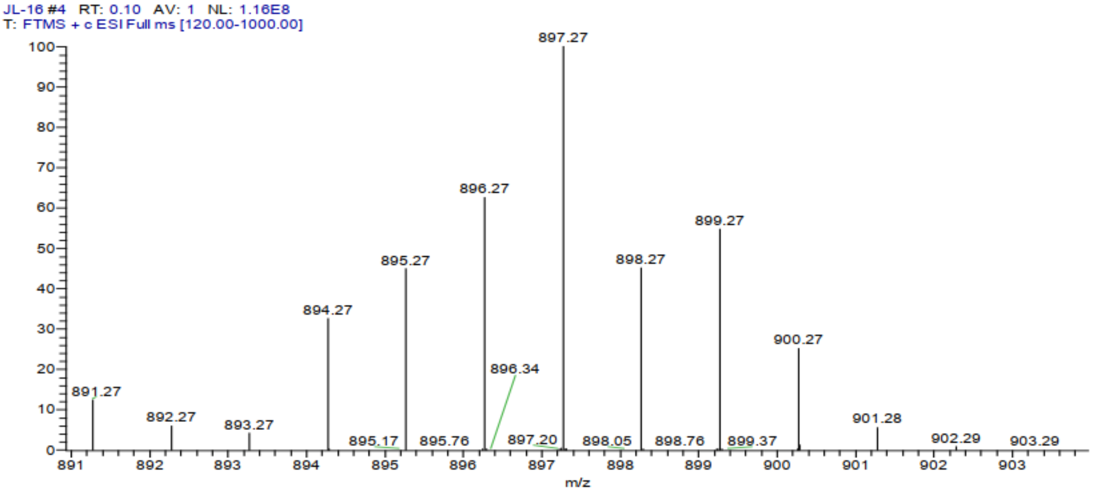
JL-16 #4 RT: 0.10 AV: 1 NL: 1.16E8
T: FTMS + c ESI Full ms [120.00-1000.00]
897.27
896.27
899.27
895.27
898.27
894.27
900.27
896.34
891.27
892.27
893.27
901.28
m/z

Complex 4

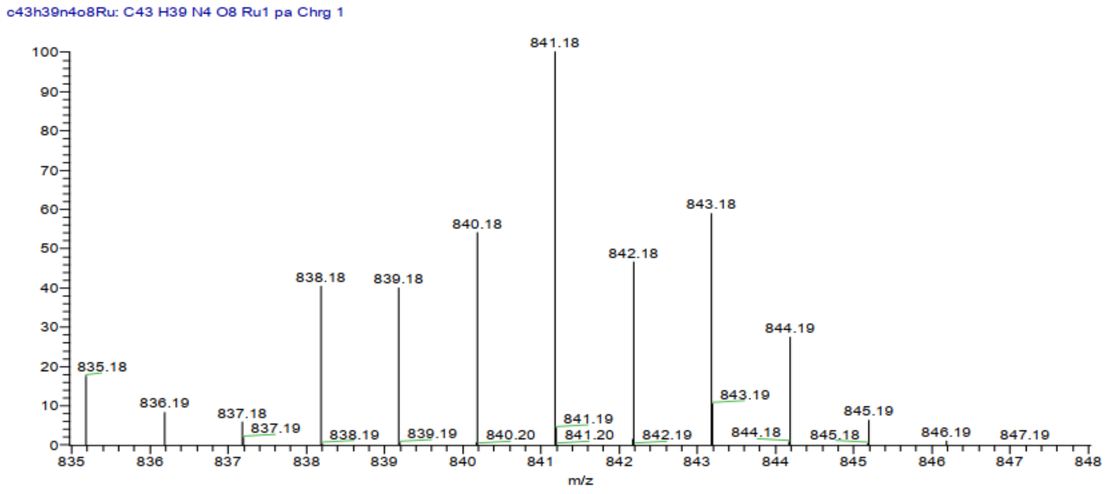
c43h39n4o8Ru: C43 H39 N4 O8 Ru1 pa Chrg 1
841.18
843.18
840.18
842.18
838.18
839.18
844.19
835.18
836.19
837.18
837.19
838.19
839.19
840.20
841.19
841.20
842.19
843.19
844.18
845.18
845.19
846.19
847.19
m/z

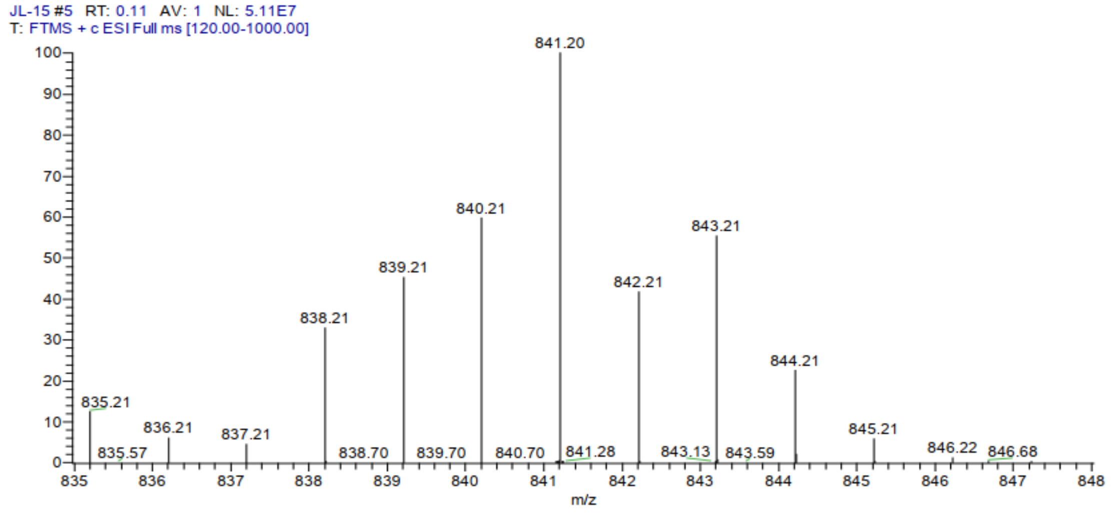
JL-15 #5 RT: 0.11 AV: 1 NL: 5.11E7
T: FTMS + c ESI Full ms [120.00-1000.00]
841.20
840.21
843.21
839.21
842.21
838.21
844.21
835.21
836.21
837.21
835.57
838.70
839.70
840.70
841.28
843.13
843.59
845.21
846.22
846.68
m/z

IR Spectra

L1 - BDMC

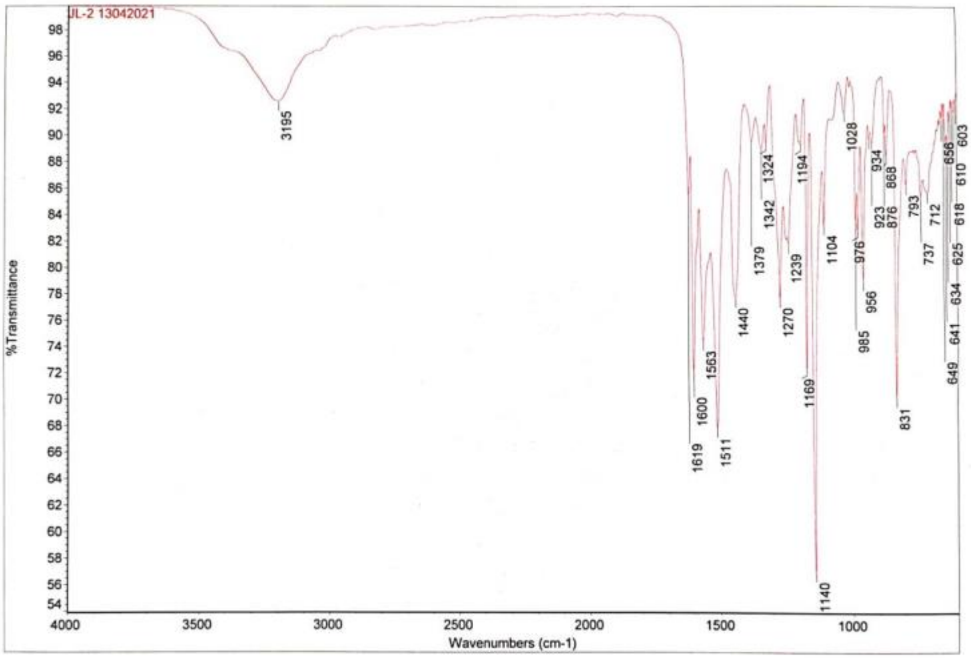

L2 - Syringaldehyde curcumin

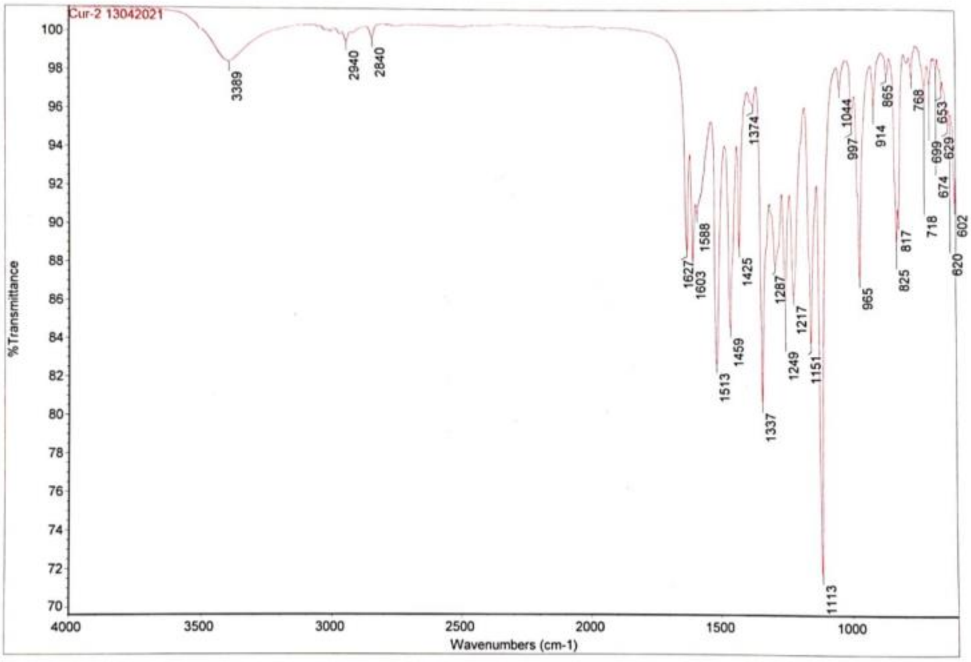

Complex **1**

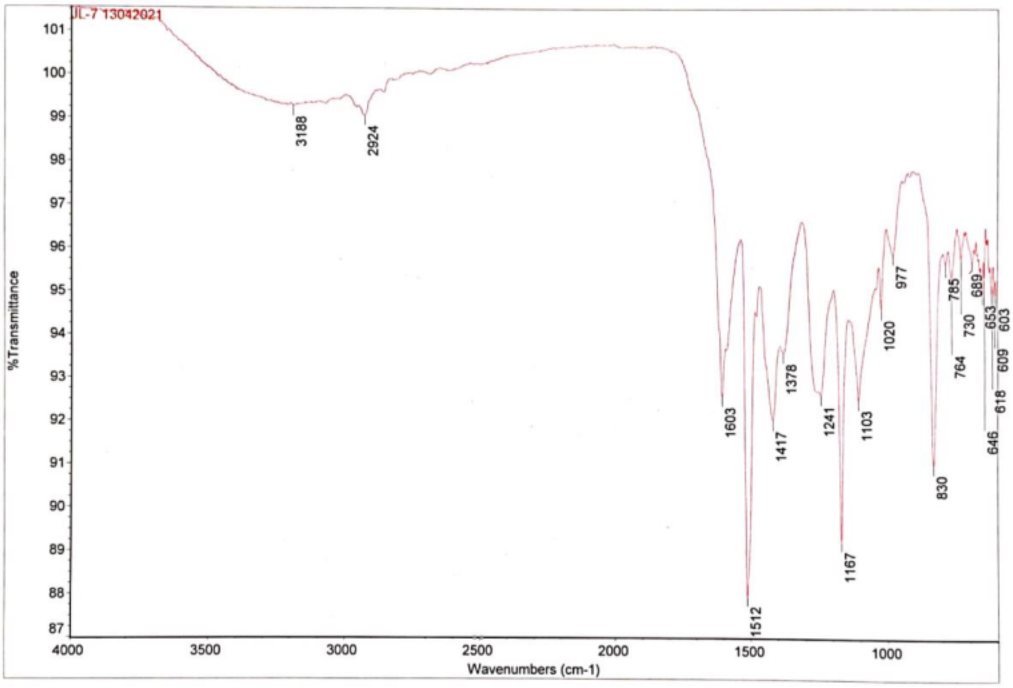

Complex **2**

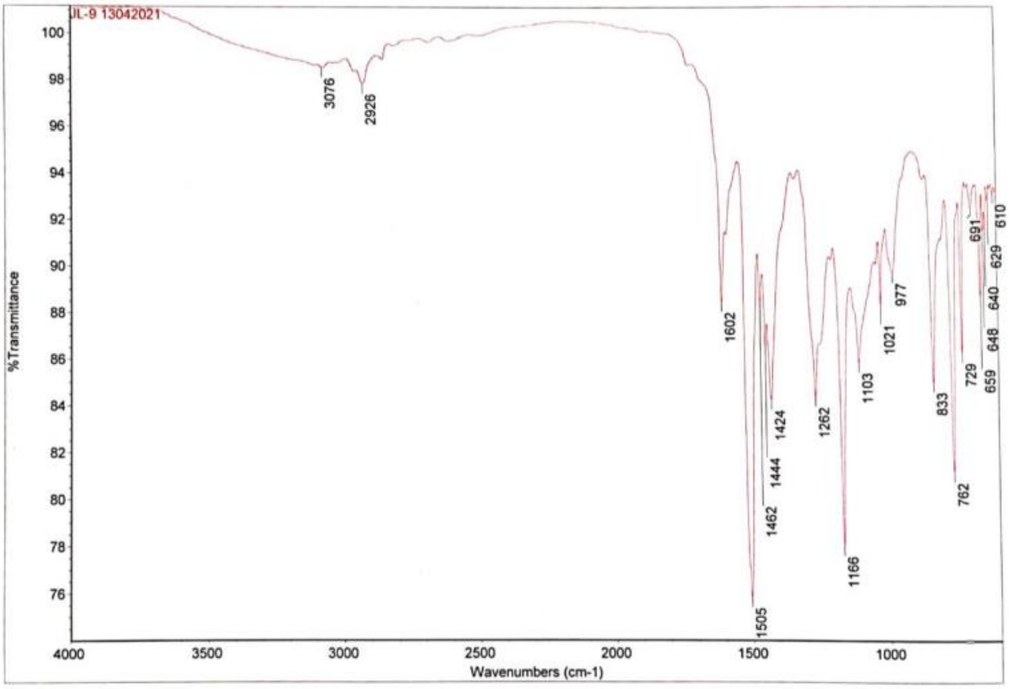

Complex **3**

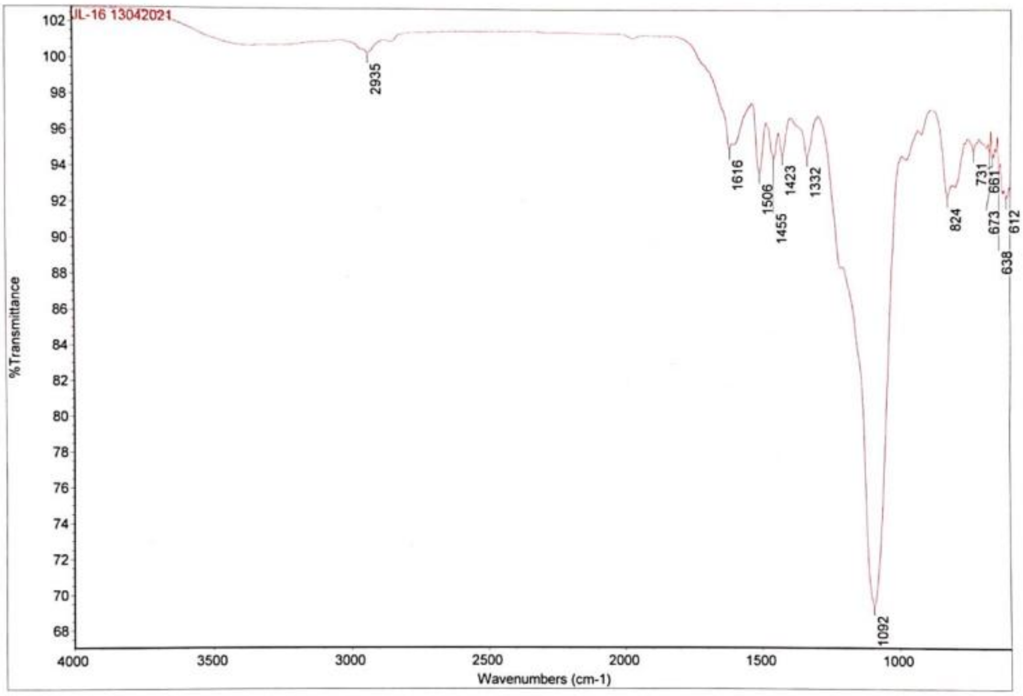

Complex **4**

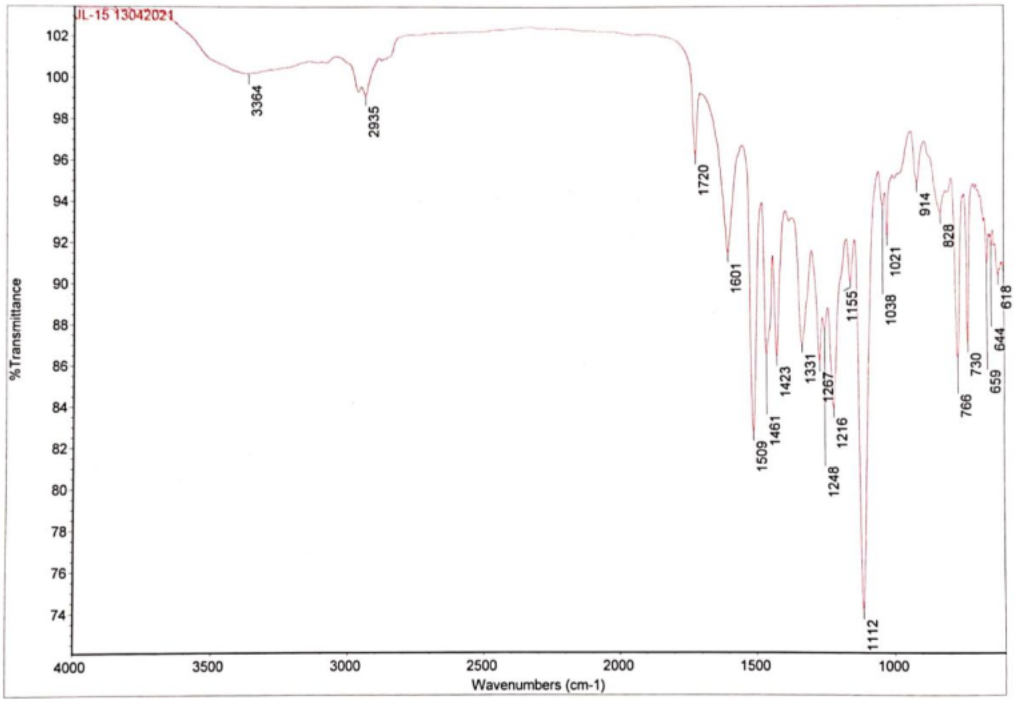

Complexes **1** and **2**

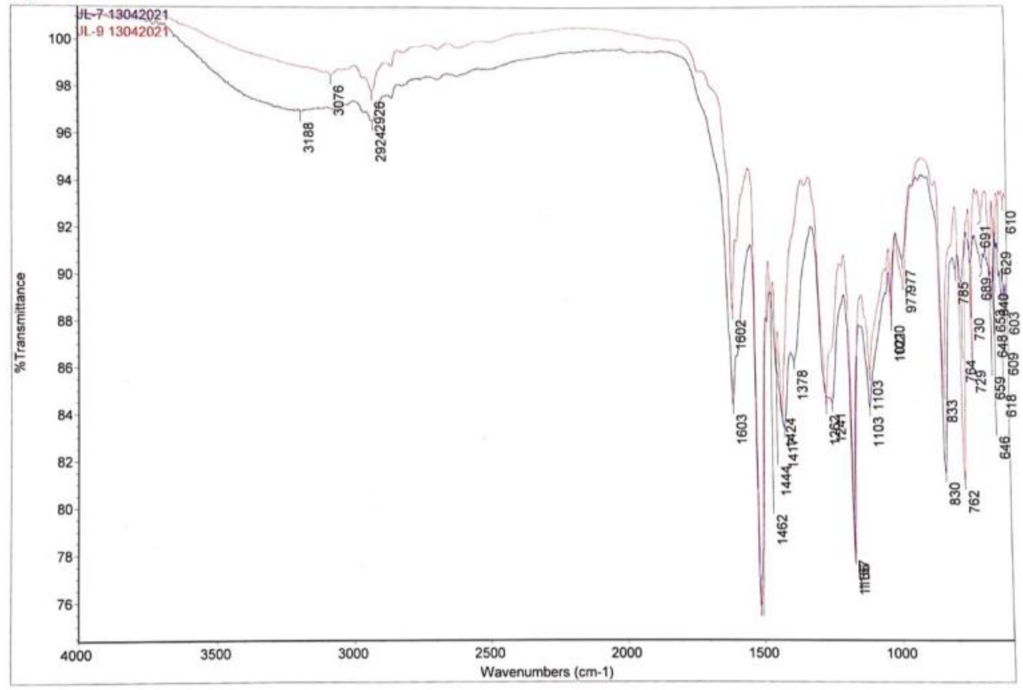

Complexes **3** and **4**

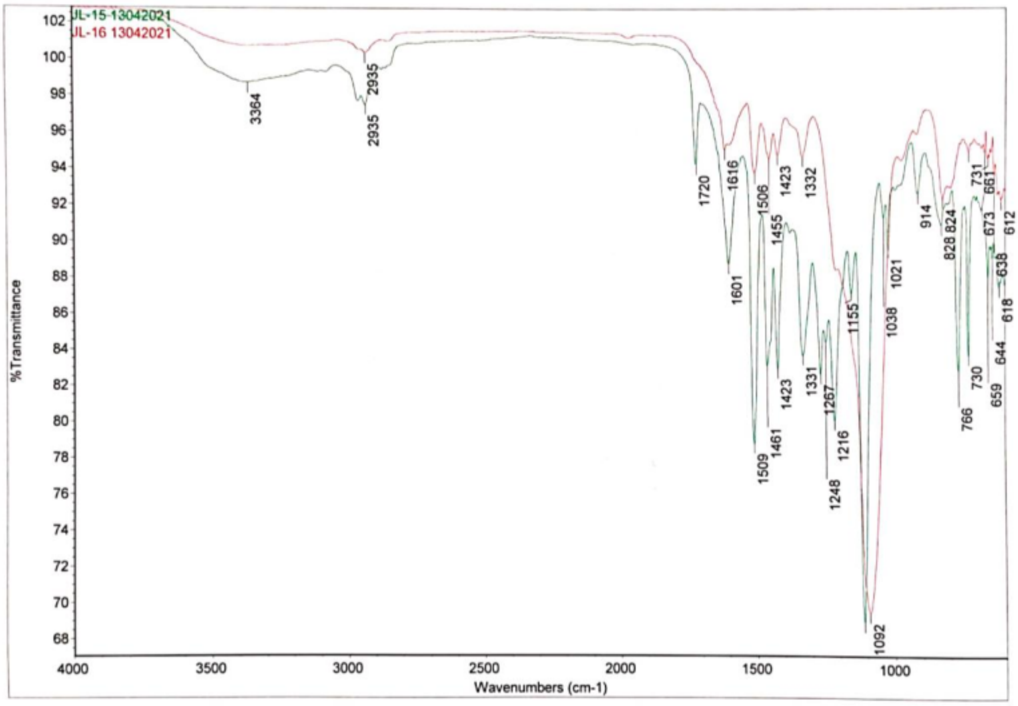

Complexes **1** and **3**

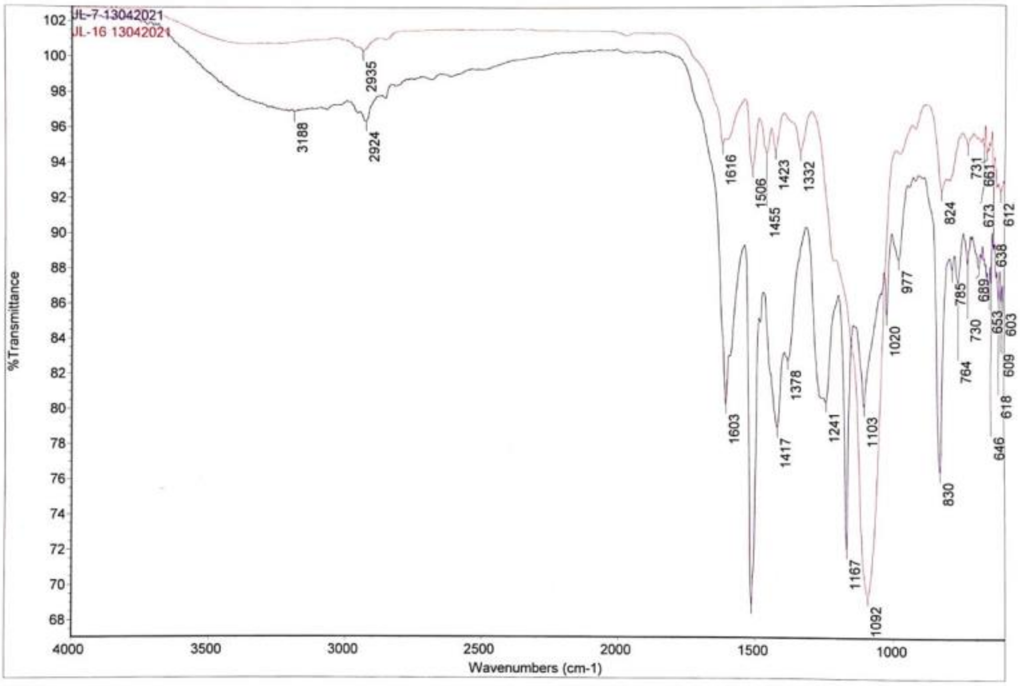

Table S1 Eigenvalues provided by the Principal component analysis, based on active variables NF-κB, FGF-2 and MMP-9, in correlation with IC50 values reflecting cytotoxicity.

Variable	Eigenvalue	% total variance	Cumulative Eigenvalue	Cumulative %
NF-κB	1.804357	45.10894	1.804357	45.1089
FGF-2	1.331217	33.28042	3.135574	78.3894
MMP-9	0.706521	17.66303	3.842095	96.0524
IC_{50}	0.157905	3.94761	4.000000	100.0000

Table S2 Correlation matrix of factor coordinates generated by PCA, corresponding to the variations of IC_{50}, NF-κB, FGF-2 and MMP-9 in A2780 cells following the time-dependent treatment with the series of novel synthesized ruthenium complexes **1-4**.

Variable	Factor 1	Factor 2	Factor 3	Factor 4
NF-κB	0.764701	0.506585	-0.325586	0.229341
FGF-2	-0.351237	0.881138	-0.241306	-0.204939
MMP-9	-0.630277	-0.387184	-0.668951	0.073109
IC_{50}	0.836046	-0.385063	-0.307882	-0.240753

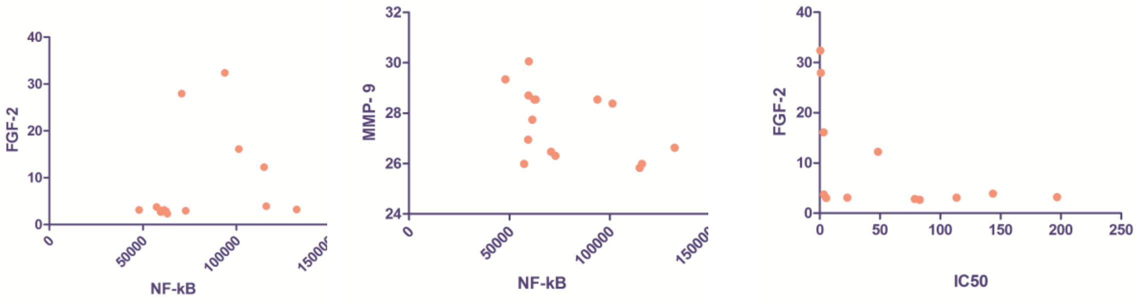

(**a**) (**b**) (**c**)

Figure S1 The intracellular activated NF-κB p65 in treated A2780 cells (**a**) correlates well with FGF-2 secreted by the cells (nonparametric Spearman correlation coefficient r = 0.4665, p value 0.0398), and (**b**) increases inversely proportional to MMP-9 (r = -0.4592, p value 0.0425). As well, it is a significant negative correlation between (**c**) the compounds cytotoxicity and FGF-2
(r = -0.5604, p value 0.0290).

Stability studies of complexes 1-4 in methanol and stock solution (ethanol:water; 1:20)

Complex 1 in methanol

```
Operator:            PC19216812\hp
Created:             13. 6. 2022 11:45:22
Spectrophotometer:   GENESYS 10S UV-Vis
Serial number:       2L9P364001
Firmware:            4.003
Baseline:            13. 6. 2022 11:45:22
```

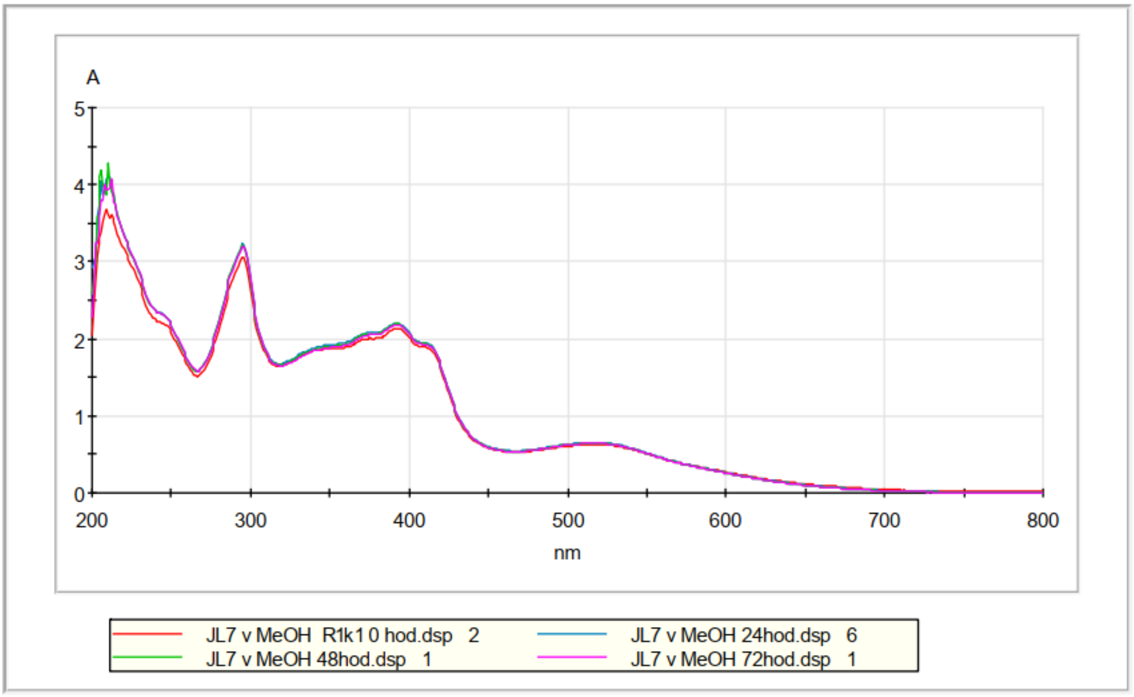

Complex 2 in Methanol

```
Operator:              PC19216812\hp
Created:               14. 6. 2022 11:55:03
Spectrophotometer:     GENESYS 10S UV-Vis
Serial number:         2L9P364001
Firmware:              4.003
Baseline:              14. 6. 2022 11:55:03
```

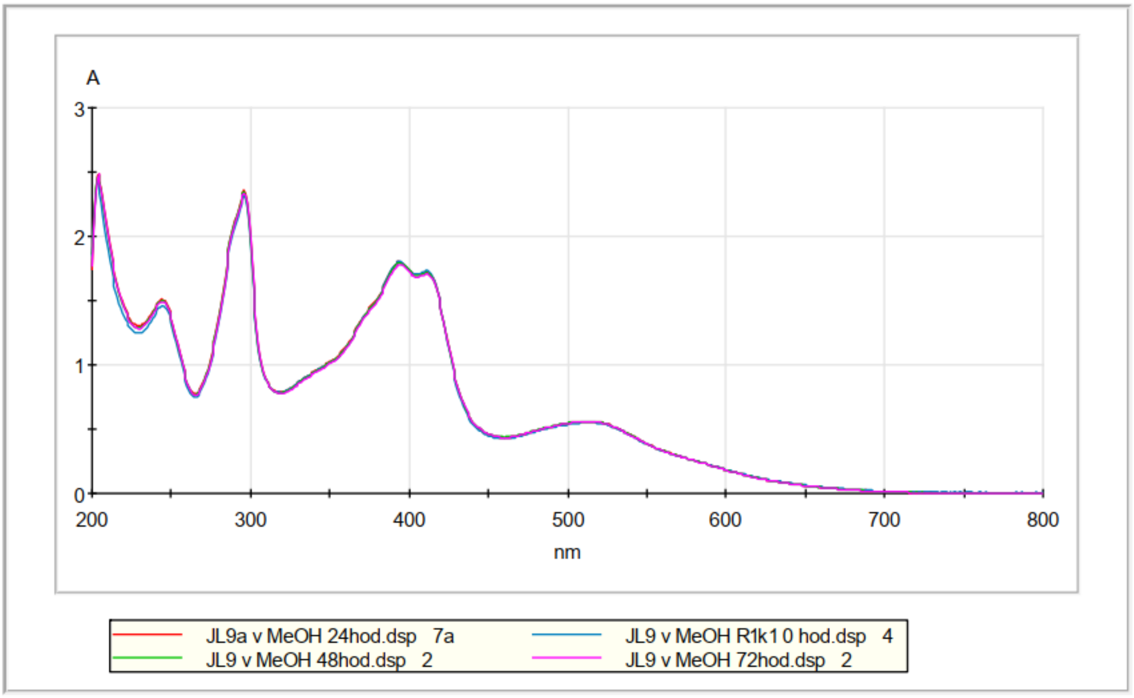

Complex 3 in Methanol

```
Operator:              PC19216812\hp
Created:               13. 6. 2022 12:06:12
Spectrophotometer:     GENESYS 10S UV-Vis
Serial number:         2L9P364001
Firmware:              4.003
Baseline:              13. 6. 2022 12:06:12
```

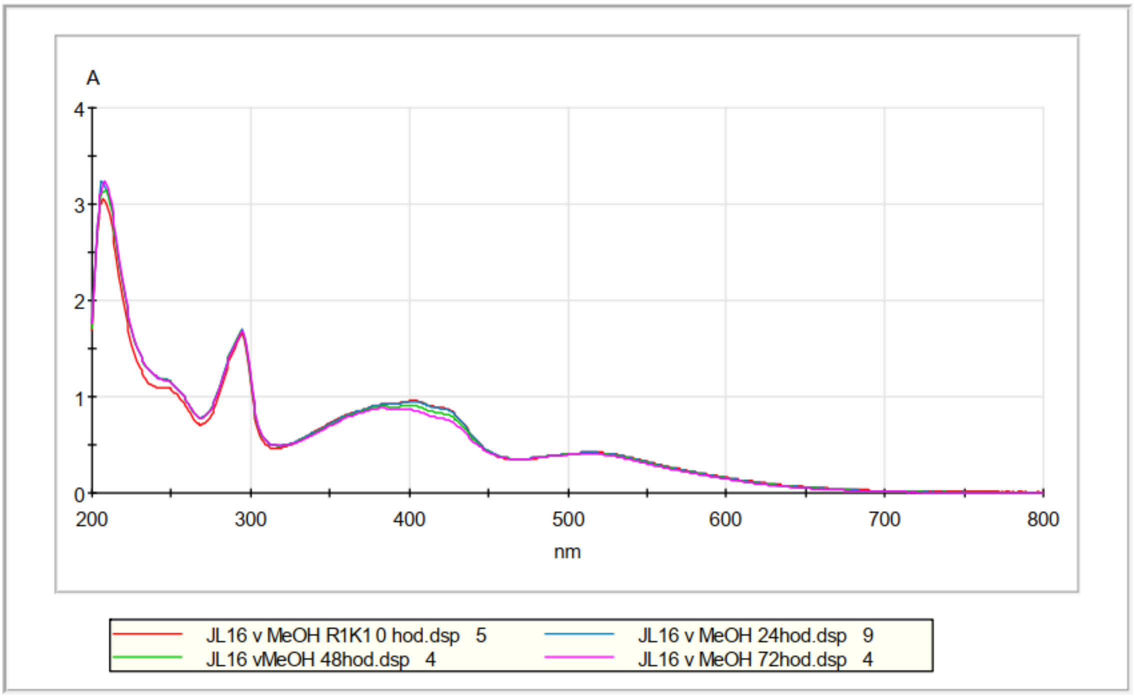

Complex 4 in Methanol

```
Operator:               PC19216812\hp
Created:                13. 6. 2022 12:01:46
Spectrophotometer:      GENESYS 10S UV-Vis
Serial number:          2L9P364001
Firmware:               4.003
Baseline:               13. 6. 2022 12:01:46
```

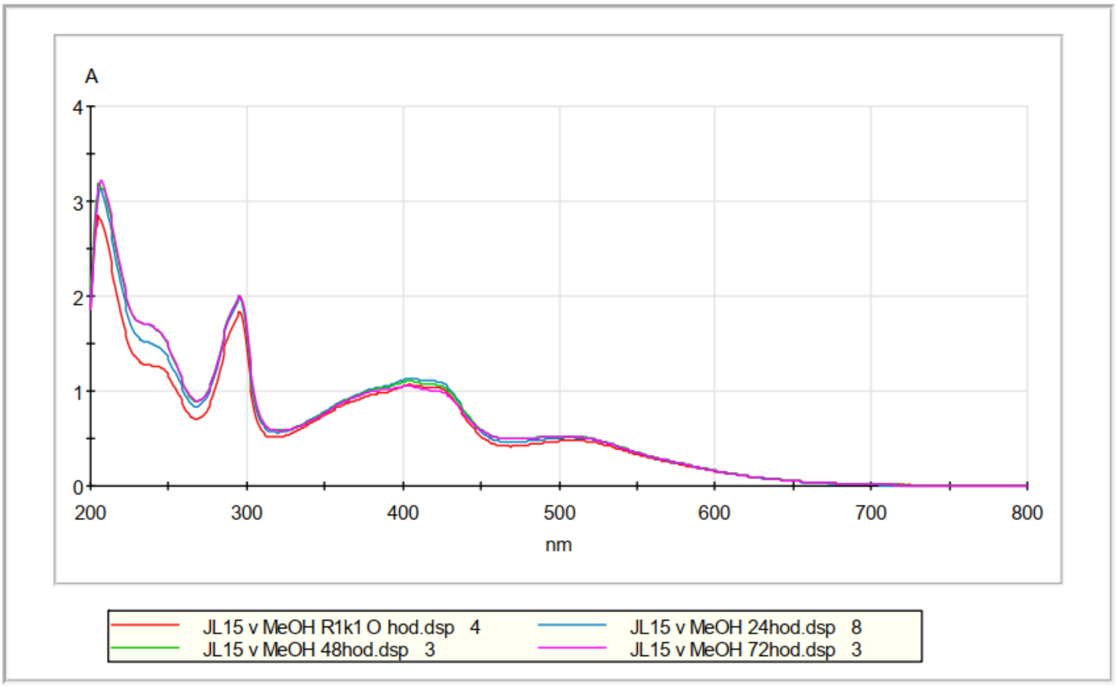

Complex 1 in ethanol + water

```
Operator:              PC19216812\hp
Created:               13. 6. 2022 12:14:49
Spectrophotometer:     GENESYS 10S UV-Vis
Serial number:         2L9P364001
Firmware:              4.003
Baseline:              13. 6. 2022 12:14:49
```

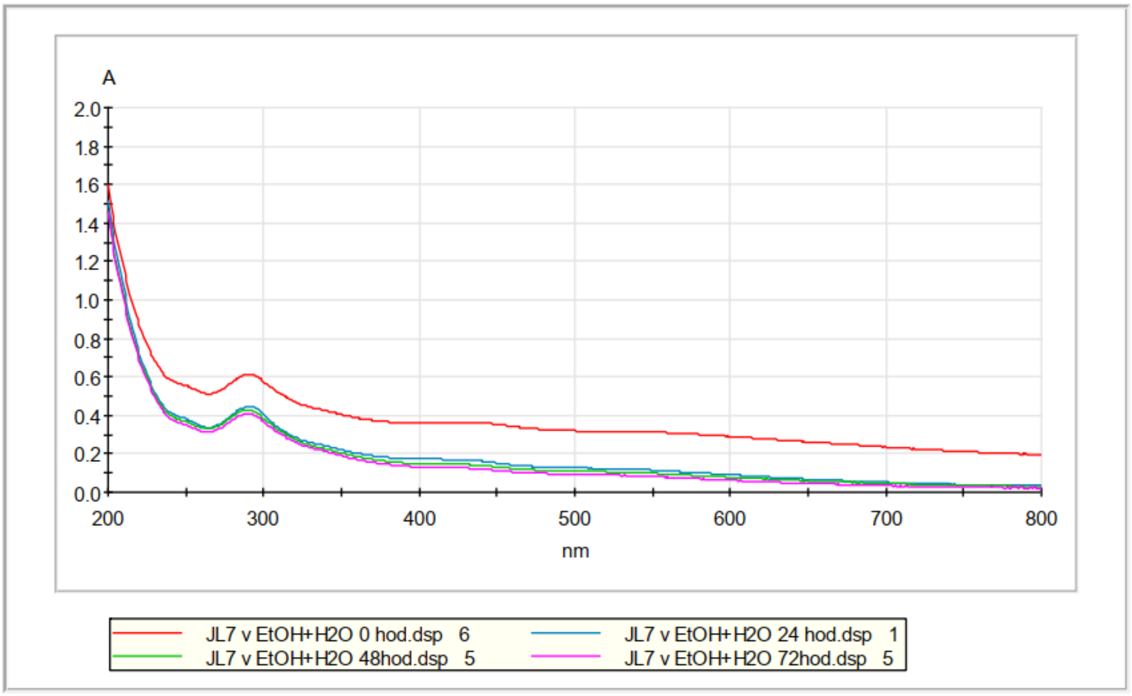

Complex 2 in ethanol + water

```
Operator:               PC19216812\hp
Created:                13. 6. 2022 12:26:36
Spectrophotometer:      GENESYS 10S UV-Vis
Serial number:          2L9P364001
Firmware:               4.003
Baseline:               13. 6. 2022 12:26:36
```

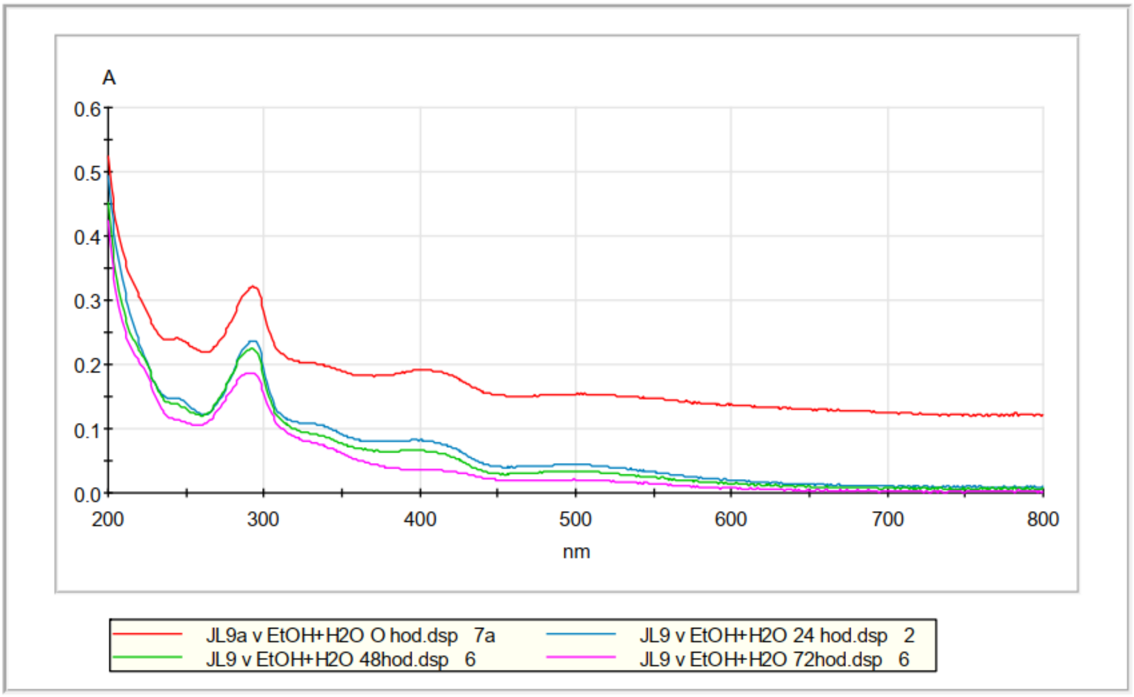

Complex 3 in ethanol + water

Operator:	PC19216812\hp
Created:	13. 6. 2022 12:34:22
Spectrophotometer:	GENESYS 10S UV-Vis
Serial number:	2L9P364001
Firmware:	4.003
Baseline:	13. 6. 2022 12:34:22

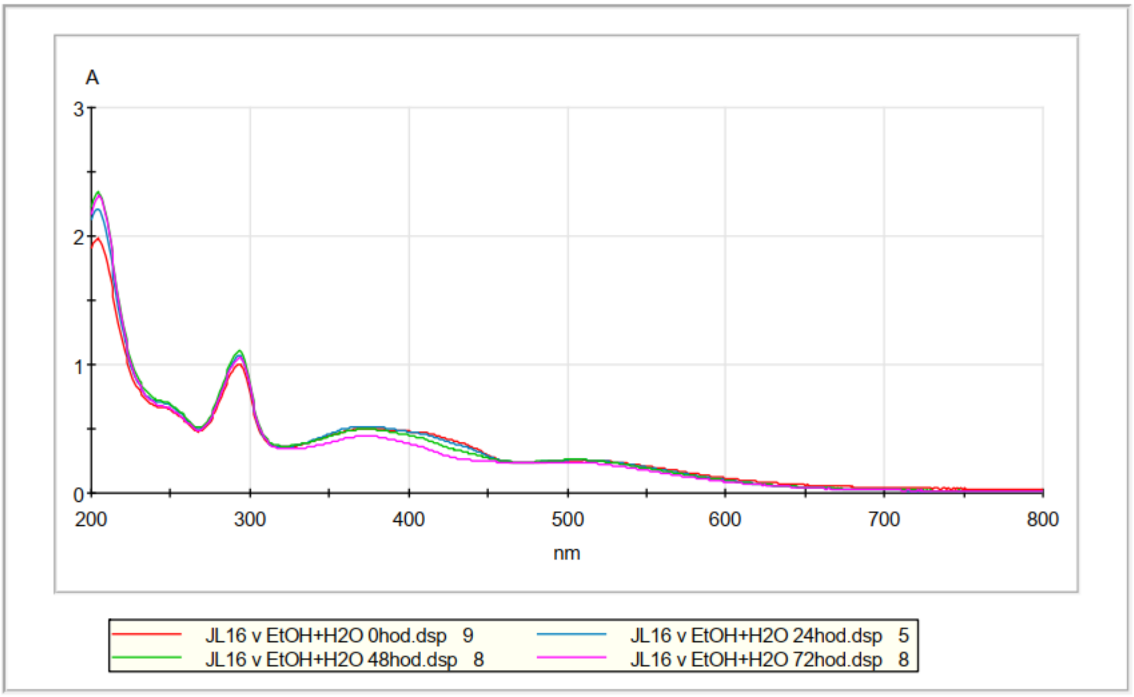

Complex 4 in ethanol + water

Spectrum:	JL15 v EtOH+H2O Ohod.dsp
Description:	8
Operator:	PC19216812\hp
Created:	13. 6. 2022 12:30:02
Spectrophotometer:	GENESYS 10S UV-Vis
Serial number:	2L9P364001
Firmware:	4.003
Baseline:	13. 6. 2022 12:30:02

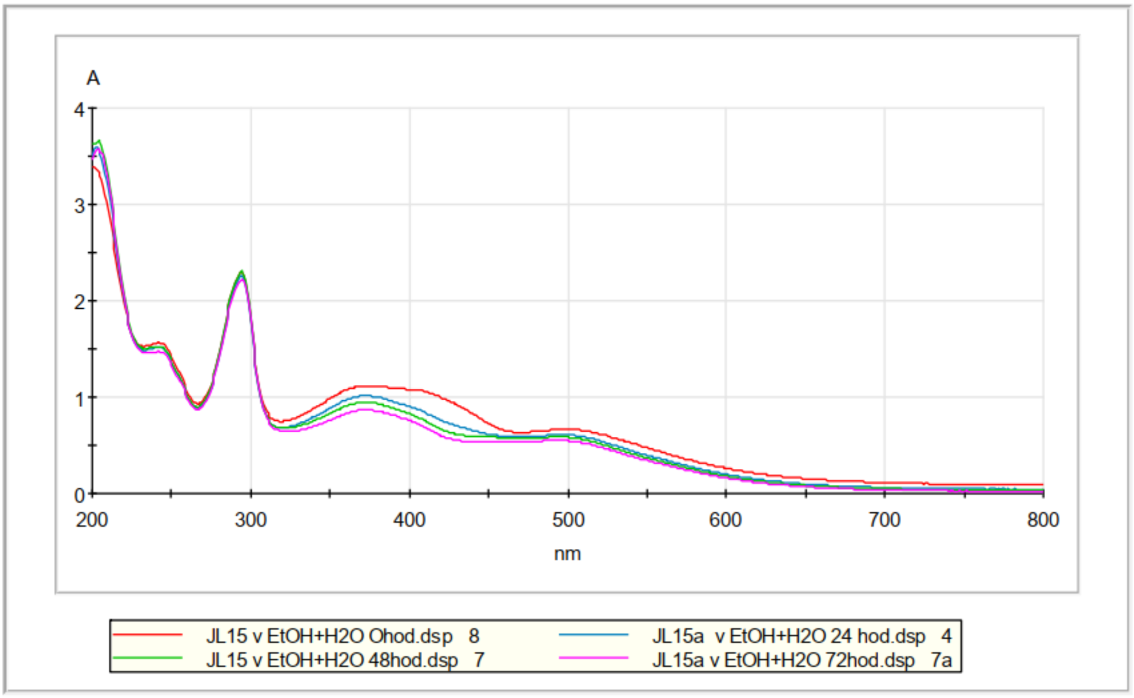

Supplement: Supplementary file 1 [file molecules-27-04565-s001.zip › molecules-1774365-supplementary.pdf]
